# Pathogenic implications of distinct patterns of iron and zinc in chronic MS lesions

**DOI:** 10.1007/s00401-017-1696-8

**Published:** 2017-03-22

**Authors:** Bogdan F. Popescu, Josa M. Frischer, Samuel M. Webb, Mylyne Tham, Reginald C. Adiele, Christopher A. Robinson, Patrick D. Fitz-Gibbon, Stephen D. Weigand, Imke Metz, Susan Nehzati, Graham N. George, Ingrid J. Pickering, Wolfgang Brück, Simon Hametner, Hans Lassmann, Joseph E. Parisi, Guo Yong, Claudia F. Lucchinetti

**Affiliations:** 1 Department of Anatomy and Cell Biology, College of Medicine, University of Saskatchewan, 701 Queen Street, Saskatoon, SK S7N 5E5 Canada; 2 Cameco MS Neuroscience Research Center, University of Saskatchewan, 701 Queen Street, Saskatoon City Hospital, Rm 5800, Saskatoon, SK S7K 0M7 Canada; 3 Department of Neurosurgery, Medical University Vienna, Vienna, Austria; 4 Stanford Synchrotron Radiation Lightsource, SLAC National Accelerator Laboratory, Menlo Park, CA USA; 5 Department of Pathology and Laboratory Medicine, Saskatoon Health Region/College of Medicine, University of Saskatchewan, Saskatoon, SK Canada; 6 Department of Health Sciences Research, Mayo Clinic, College of Medicine, Rochester, MN USA; 7 Department of Neuropathology, University of Göttingen, Göttingen, Germany; 8 Molecular and Environmental Science Research Group, Department of Geological Sciences, University of Saskatchewan, Saskatoon, Canada; 9 Toxicology Center, University of Saskatchewan, Saskatoon, Canada; 10 Department of Chemistry, University of Saskatchewan, Saskatoon, Canada; 11 Department of Neuroimmunology, Center for Brain Research, Medical University of Vienna, Vienna, Austria; 12 Department of Laboratory Medicine and Pathology, Mayo Clinic, Rochester, MN USA; 13 Department of Neurology, Mayo Clinic, College of Medicine, 200 First Street SW, Rochester, MN 55905 USA

**Keywords:** Synchrotron, Iron, Zinc, Multiple sclerosis, Oligodendrocyte, Smoldering lesion, Shadow plaque, Astrocyte, Remyelination

## Abstract

Multiple sclerosis (MS) is a chronic inflammatory demyelinating disease of the central nervous system (CNS) in which oligodendrocytes, the CNS cells that stain most robustly for iron and myelin are the targets of injury. Metals are essential for normal CNS functioning, and metal imbalances have been linked to demyelination and neurodegeneration. Using a multidisciplinary approach involving synchrotron techniques, iron histochemistry and immunohistochemistry, we compared the distribution and quantification of iron and zinc in MS lesions to the surrounding normal appearing and periplaque white matter, and assessed the involvement of these metals in MS lesion pathogenesis. We found that the distribution of iron and zinc is heterogeneous in MS plaques, and with few remarkable exceptions they do not accumulate in chronic MS lesions. We show that brain iron tends to decrease with increasing age and disease duration of MS patients; reactive astrocytes organized in large astrogliotic areas in a subset of smoldering and inactive plaques accumulate iron and safely store it in ferritin; a subset of smoldering lesions do not contain a rim of iron-loaded macrophages/microglia; and the iron content of shadow plaques varies with the stage of remyelination. Zinc in MS lesions was generally decreased, paralleling myelin loss. Iron accumulates concentrically in a subset of chronic inactive lesions suggesting that not all iron rims around MS lesions equate with smoldering plaques. Upon degeneration of iron-loaded microglia/macrophages, astrocytes may form an additional protective barrier that may prevent iron-induced oxidative damage.

**Electronic supplementary material:**

The online version of this article (doi:10.1007/s00401-017-1696-8) contains supplementary material, which is available to authorized users.

## Introduction

Multiple sclerosis (MS) is a central nervous system (CNS) chronic inflammatory demyelinating disease which targets oligodendrocytes and myelin. Oligodendrocytes are the CNS cells that stain most robustly for iron [63], and are also highly susceptible to inflammatory-mediated injury [73]. Metals are essential for the synthesis, stability, and maintenance of myelin [14, 29, 36, 63, 64], and are required for normal CNS functioning [36, 72]. Metal dyshomeostasis causes myelin breakdown in Huntington disease [4], and hyperzincemia-induced copper deficiency [51]. This suggests an important role for iron and other metals, such as zinc, in MS pathogenesis. Iron levels in particular must be balanced delicately since loading of cells with iron may lead to the formation of reactive oxygen species and oxidative damage of DNA, lipids, and proteins.

Oxidative stress, dysregulation of metals and metalloproteins in the serum, cerebrospinal fluid or brains of MS patients, and iron liberation in the CNS extracellular space have been linked to the conversion of isolated demyelinating episodes to clinically definite MS, as well as to MS progression via amplification of demyelination and neurodegeneration [22, 27, 31, 56]. While some magnetic resonance imaging (MRI) sequences have been used to image iron in vivo in MS [1, 3, 26, 39, 75, 76], histochemistry is the gold standard to localize metals in tissue. However, iron histochemistry detects only nonheme iron [38], and histochemistry for zinc has poor sensitivity, specificity and is complex [15, 32]. Each method employs a different chemical reaction, and therefore cannot be used on the same tissue section. X-ray fluorescence imaging (XFI) is element-specific, quantitatively detects all chemical forms of any metal and simultaneously maps multiple metals, therefore addressing the limitations of histochemistry [45, 46, 48, 49, 52]. This is the first systematic synchrotron X-ray fluorescence study to compare the distribution and quantification of iron and zinc in MS lesions to the surrounding normal appearing white matter (normal appearing WM) and periplaque white matter (periplaque WM) from a given patient, and to assess the involvement of these metals in MS lesion pathogenesis.

## Patients and methods

### Sample characterization

Study approval was granted by the University of Saskatchewan Biomedical Research Ethics Board (Bio# 11-217), Mayo Clinic Institutional Review Board (IRB-2067-99), and University Medical Center Göttingen ethical review committee (14/05/03). We analyzed formalin-fixed paraffin-embedded archival autopsy tissue from 18 MS patients (Suppl. Table 1), with no known iron metabolism abnormalities. Tissue was embedded in paraffin at autopsy under routine neuropathology processing, therefore limiting variability between cases with respect to fixation duration. Clinical diagnosis was determined according to McDonald or Poser criteria by a certified neurologist [37, 50]. Clinical course was defined as acute monophasic MS resulting in death within a year, relapsing remitting (RR) MS, secondary progressive (SP) MS, primary progressive (PP) MS, or uncertain, when insufficient clinical data rendered sub-classification not possible [33].

### Neuropathology and Immunohistochemistry

A certified neuropathologist confirmed the pathological diagnosis of inflammatory demyelination consistent with MS. Microscopic sections, 5-μm thick, were stained with hematoxylin and eosin for morphological evaluation, Luxol fast blue (LFB)/periodic acid–Schiff or LFB/hematoxylin & eosin for myelin, and silver impregnation for axons. Iron histochemistry was performed using a DAB-enhanced Turnbull staining that detects all nonheme ferric and ferrous iron [38], and is therefore the appropriate complementary method that enables a direct comparison to XFI. Immunohistochemistry was performed using an avidin–biotin technique using the primary antibodies listed in Suppl. Table 2.

White matter plaques were classified as described previously [19]. Smoldering plaques showed an inactive demyelinated center surrounded by a rim of activated microglia/macrophages containing myelin debris [18]. Inactive plaques were completely demyelinated with a sharp border and few macrophages/microglia. Completely remyelinated (shadow) plaques were sharply circumscribed regions of reduced myelin staining. Periplaque and normal appearing WM referred to the white matter immediately surrounding the demyelinated lesion, or white matter located at least 2 cm away from the lesion, respectively.

Cortical demyelinated lesions were classified as described previously [28, 34]. Leukocortical lesions involved both the deeper cortex and subjacent white matter. Intracortical lesions were centered on blood vessels and confined within the cortex. Subpial lesions extended from pia into the deeper cortical layers.

### Synchrotron X-ray fluorescence imaging

Synchrotron XFI was performed at the Stanford Synchrotron Radiation Lightsource (SSRL), Stanford University, Menlo Park, CA, USA on 15-μm thick sections collected on metal-free plastic coverslips (Nunc Thermanox, Thermo Scientific, USA). Whole sections were imaged on beamline 10-2, mounted at 45° to the incident beam (12.5 keV) which passed through a glass polycapillary (XOS, USA) to produce a 50 μm × 50 μm spot on the sample. The beam exposure time was 80 ms/pixel. Fluorescent energy windows were centered for iron (6.21–6.70 keV) and zinc (8.38–8.98 keV).

Regions of interest (ROI) were imaged at higher resolution using the microfocused XFI on beamline 2–3 with a 3 μm × 3 μm incident X-ray beam (7.15 keV) focused with Kirkpatrick–Baez mirrors, and a 120 ms/pixel dwell time. Fluorescence was normalized against the incident X-ray beam intensity to take into account its fluctuations. X-ray fluorescence was detected using a silicon drift detector (Hitachi) mounted 90° to the incident beam utilizing the Xpress3 signal processing system (Quantum Detectors, UK).

### Histology: XFI correlation and XFI data analysis

Sections stained for proteolipid protein were scanned at 40× magnification using an Olympus VS110 slide scanner. Whole maps were saved as JPEG files on which normal appearing and periplaque WM, normal cortex, and lesion ROIs were outlined. XFI images were generated using MicroTool Kit software [68]. Histology and XFI maps were displayed side by side, and XFI ROIs were outlined using the histology maps as guides (Suppl. Fig. 1). Normalized X-ray fluorescence was obtained for each ROI pixel. Data were then imported into R for statistical analysis (R Foundation for Statistical Computing, Vienna, http://R-project.org). The impact of blood metals from large vessels was accounted for by excluding them from the ROI at the time of quantification.

### X-ray absorption near edge structure

X-ray absorption near edge structure (XANES) spectra of ROIs (Suppl. Fig. 2) detected during microfocused XFI were acquired on beamline 2–3 equipped with a Si(111) double-crystal monochromator from one smoldering and one inactive lesion from different patients. The incident and transmitted X-ray intensities were monitored using N_2_-filled ionization chambers. The Fe K-edge spectra were recorded as K fluorescence excitation spectra using a silicon drift detector. The X-ray energy was calibrated to the lowest K-edge energy inflection point of a standard iron foil (EXAFS Co), whose energy was assumed to be 7111.3 eV. Five to seven points of interest (each encompassing a 7 μm^2^ area) were randomly sampled from within each plaque subregion and averaged to representatively assess the chemical form of iron in each subregion (Suppl. Fig. 2). Eight to sixteen sweeps were collected for each point of interest and averaged to improve the signal to noise ratio. Therefore, each spectrum in Figs. 4, 6 and Table 2 represents an average of 40–90 spectra collected from 5 to 7 different points of interest (Suppl. Fig. 2).

### X-ray absorption near edge structure data analysis

Data analysis was performed using the EXAFSPAK computer program suite (http://ssrl.slac.stanford.edu/exafspak.html). Quantitative determination of the iron chemical forms was carried out by least-squares fitting of near-edge spectra to linear combinations of spectra of different standards (Suppl. Table 3) [43, 47]. No smoothing or related data manipulations were performed.

### Statistical analysis

We used a two-stage statistical approach in analyzing normalized X-ray fluorescence counts. First, we calculated the median normalized metal counts across all pixels in a given ROI. The median was used as a measure of central tendency that was robust to right skew and possible outliers. Second, we fit a linear mixed effects regression model using the lme4 package in R with normalized counts as the response and treating 7-level region type as a random effect. This served to shrink estimates of the normalized counts in each region type towards the overall average and control false positives [20]. The mixed effects models also included a subject-specific random intercept to account for intraclass correlation among regions from the same individual. Additional analyses examined the effects of age and duration using mixed models with a random age by region type or duration by region type interaction. These models allowed for separate associations with age or duration for each region type that were shrunk towards the overall average. Quantile-based confidence intervals (CIs) were obtained from parametric bootstrap sampling. For subset analyses involving only a few subjects we calculated a mean normalized intensity for each person averaging over all relevant regions for that individual. We then performed paired *t* tests.

## Results

### Cohort characterization

Tissues from 18 patients [ten female (56%) and eight male patients (44%)] were studied (Suppl. Table 1). The median disease duration was 16 years (range 1–42). The median age at death was 66 years (range 19–82). Two patients (11%) had RRMS, four (22%) SPMS with attacks, six (33%) SPMS without attacks, one (6%) PPMS, and one (6%) acute monophasic MS. The clinical course was unavailable in four (22%).

Twenty-one blocks containing 148 ROIs were analyzed. There were 59 lesions: 19 inactive white matter lesions (13%), ten smoldering lesions (7%), five shadow plaques (3%) and 25 cortical plaques (17%). The remaining 89 ROIs included nine regions of normal appearing WM, 35 of periplaque WM, and 45 of normal appearing cortex (Table 1).

**Table 1:** The table shows the number of subjects, blocks, and plaques with each region type

Region type, number (%)	Subjects (*n* = 18)	Blocks (*n* = 21)	Regions of interest (*n* = 148)
White matter tissue			
Normal appearing	5 (28%)	6 (29%)	9 (6%)
Periplaque	18 (100%)	21 (100%)	35 (24%)
Inactive plaque	10 (56%)	11 (52%)	19 (13%)
Smoldering plaque	8 (44%)	9 (43%)	10 (7%)
Shadow plaque	4 (22%)	4 (19%)	5 (3%)
Cortical tissue			
Normal appearing	16 (89%)	18 (86%)	45 (30%)
Leukocortical plaque	2 (11%)	2 (10%)	3 (2%)
Subpial plaque	8 (44%)	9 (43%)	20 (13%)
Intracortical plaque	2 (11%)	2 (10%)	2 (1%)

### Relationship between metals and age/disease duration in MS

Overall, iron levels decreased with increasing patient age (*p* = 0.03), irrespective of plaque type. For example, average iron levels in patients age 60 at death were an estimated 15% lower than those age 45 at death (95% CI, 2–26% lower). There was some variation in the iron vs. age relationship by region type (Suppl. Fig. 3a), although this is suggestive only since the statistical evidence for differences in the correlation by region type is limited. Patients with longer disease duration tended to have lower iron across regions, although this association did not reach statistical significance (*p* = 0.15) (Suppl. Fig. 4a). There was no evidence that zinc varied with age (*p* = 0.66) (Suppl. Fig. 3b) or disease duration (*p* = 0.49) (Suppl. Fig. 4b).

### Iron content of MS lesions

**Fig. 1:**
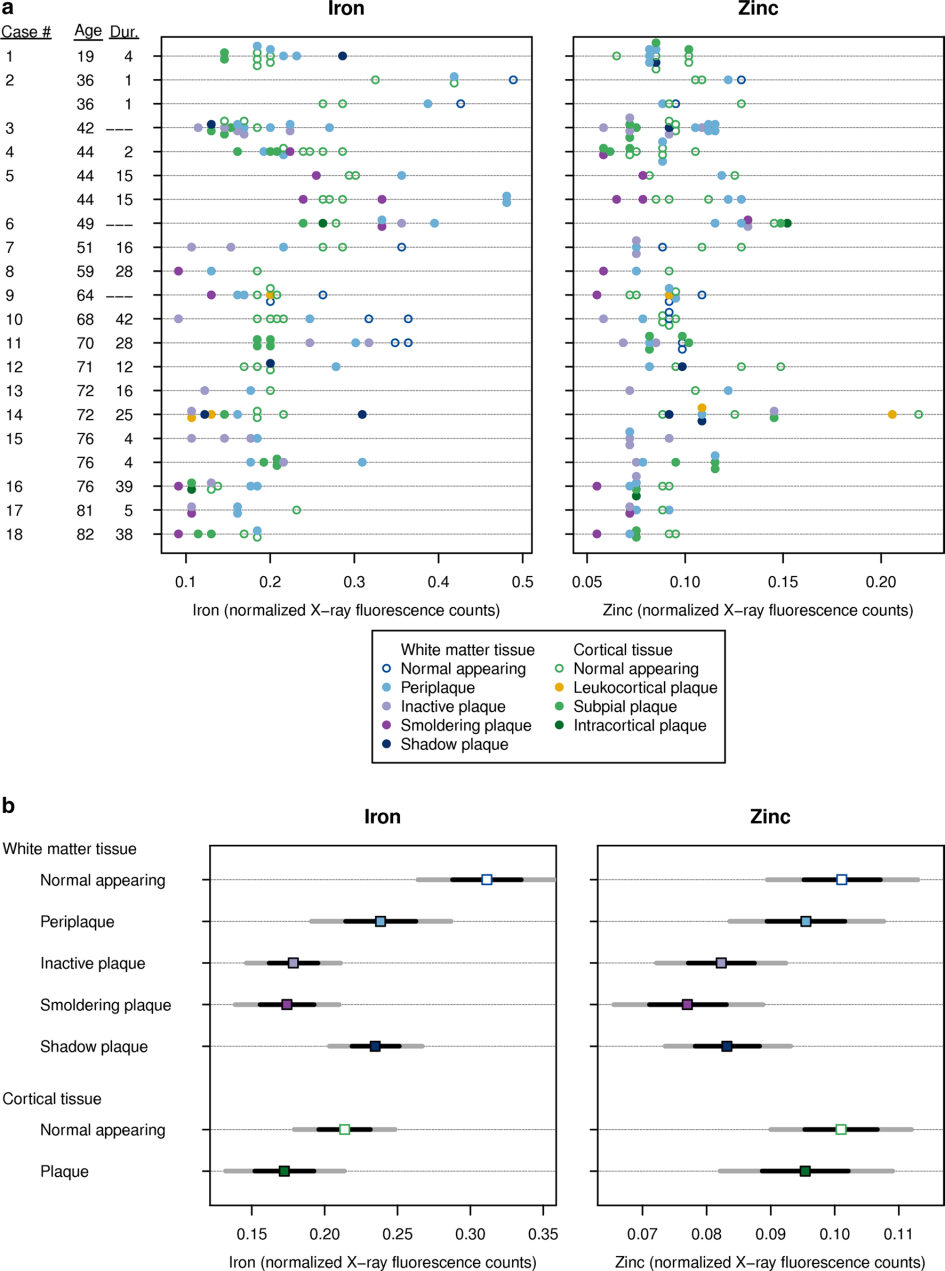
Iron and zinc in MS lesions; **a** Distribution of Fe and Zn normalized X-ray fluorescence values for each of the 21 blocks in the study. *Each point* represents one region of interest (ROI) on a particular block. The value displayed is the median metal divided by the incident X-ray beam intensity over the entire ROI multiplied by 100. The patient age at death (years) and disease duration (years) are provided on the *y*-axis, noting that three patients have two blocks; **b** Estimated metal content with 95% confidence interval (CI) (*dark gray lines*) and 68% CI (*black lines*) by plaque type. The two CIs represent ±2 SE and ±1 SE, respectively. Estimates were derived from linear mixed effects models

In the white matter, on average the normal appearing WM had the highest iron content, followed by the periplaque WM, shadow plaques, inactive and smoldering lesions (Fig. 1a, b, Suppl. Fig. 4c). Iron in normal appearing WM was significantly greater than in the periplaque WM (*p* < 0.001) and all lesion types (*p* = 0.006 vs. shadow plaques; *p* < 0.001 vs. smoldering lesions; *p* < 0.001 vs. inactive lesions). The iron content was similar between periplaque WM and shadow plaques (*p* = 0.89), and was significantly increased in periplaque WM and shadow plaques when compared to smoldering (*p* = 0.02; *p* < 0.001) or inactive lesions (*p* = 0.03; *p* < 0.001). Inactive and smoldering lesions had similar iron levels (*p* = 0.74).

In the gray matter, the normal appearing cortex had significantly elevated iron compared to cortical lesions (*p* = 0.02). (Fig. 1a, b, Suppl. Fig. 4c). The small number of leukocortical and intracortical lesions precluded any definitive conclusions regarding their iron content.

### Zinc content of MS lesions

In the white matter, the normal appearing WM had the most zinc, followed by the periplaque WM, shadow plaques, inactive and smoldering lesions (Fig. 1a, b, Suppl. Fig. 4c). The normal appearing WM had significantly increased zinc when compared to all lesion types (*p* = 0.008 vs. shadow plaques; *p* = 0.001 vs. smoldering lesions; *p* = 0.005 vs. inactive lesions). The difference between the normal appearing and periplaque WM was not significant (*p* = 0.29). Zinc in the periplaque WM was significantly higher than in smoldering lesions (*p* = 0.01), and to a lesser degree versus other lesion types (*p* = 0.06 vs. shadow plaques; *p* = 0.06 vs. inactive lesions). Zinc in inactive lesions was similar to smoldering (*p* = 0.31) and shadow plaques (*p* = 0.82). There was no significant difference between the zinc content of the smoldering and shadow plaques (*p* = 0.23).

In the gray matter, there were no significant zinc differences between the normal appearing cortex and cortical lesions (*p* = 0.39) (Fig. 1a, b, Suppl. Fig. 4c). Given the small number of leukocortical and intracortical lesions, no definitive conclusion regarding their zinc content could be determined.

### Plaque-specific metal distribution subregion patterns

#### Smoldering white matter plaques

**Fig. 2:**
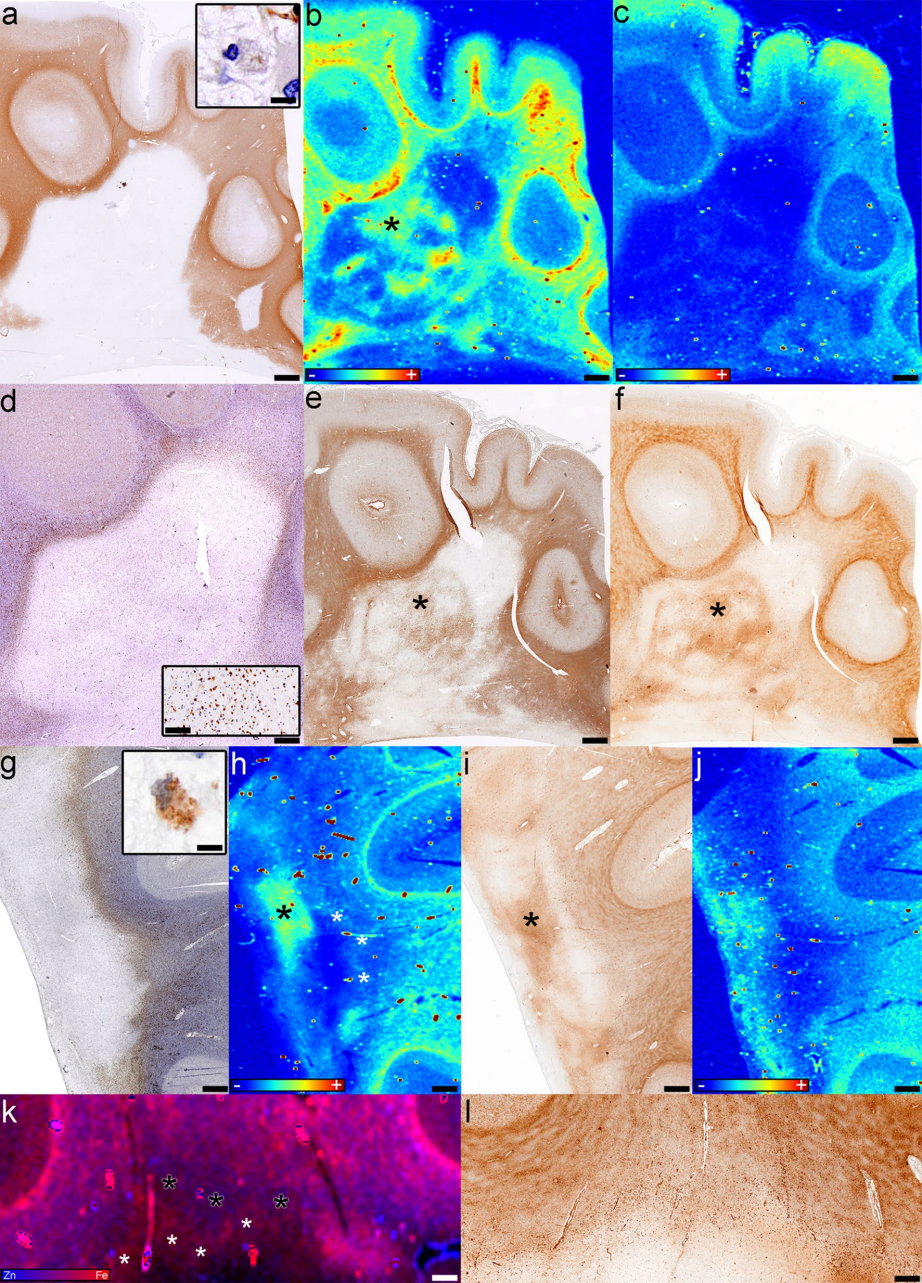
Fe and Zn in smoldering plaques. **a**–**f** Case 5, block 2 in Fig. 1a: **a** Demyelination is seen as lack of PLP immunoreactivity; inset shows macrophages in the smoldering rim containing myelin debris (PLP); **b** Fe accumulates in the inactive center of smoldering lesions (*asterisk*) (XFI); **c** The demyelinated lesion lacks Zn (XFI); **d** Macrophages/microglia accumulate at the edge of the smoldering lesion; *inset* shows higher magnification of the smoldering rim (CD68); **e** Reactive astrocytes and fibrillary gliosis are present in the inactive center (*asterisk*) (GFAP); **f** Fe accumulates within the inactive center of smoldering lesions (*asterisk*) (Fe histochemistry); **g**–**l** Case 4 in Fig. 1a: **g** The rim of macrophages/microglia is seen at the smoldering plaque’s edge (CD68); *inset* shows that macrophages in the smoldering rim contain myelin debris (PLP); **h** Iron accumulates in the inactive center (*black asterisk*) and to a lesser extent in the rim (*white asterisks*) (XFI); **i** The Turnbull stain also shows iron accumulation in the inactive center (*black asterisk*) (Fe histochemistry); **j** Zn is located periventricularly (XFI); **k** The overlay of Fe and Zn shows the iron-accumulating rim of the smoldering plaque (*white asterisks*) and the iron-poor periplaque WM (*black asterisks*) (XFI); **l** The Turnbull stain does not show the iron-poor periplaque WM (Fe histochemistry); *Scale bars* **a**–**j** 3 mm; *Scale bars* insets **a**, **j** 25 μm; *Scale bar* inset **d** 200 μm; *Scale bars* **k**, **l** 1 mm. *Color scales* **b**, **c**, **h**, **j** represent the normalized total Kα fluorescence counts, proportional to total metal present, from *blue* (lowest) to *red* (highest); *Color scale* **k** represents the overlay of the normalized total Fe and Zn Kα fluorescence counts, proportional to total metal present, from *blue* (Zn) to *red* (Fe)

We identified ten smoldering plaques (Table 1; Fig. 2a, d, g) from nine tissue blocks from eight patients (four SPMS, three without and one with attacks; one RRMS; three with uncertain clinical course) characterized by a variable degree of demyelinating activity (Fig. 2a, g insets) at the rim. Three smoldering plaques demonstrated a dense myelin-laden macrophage accumulation, whereas seven had few macrophages containing myelin debris at the rim. The rim of macrophages/microglia (Fig. 2d, g) in 4/10 smoldering plaques contained iron visible on XFI (Figs. 2h, k, 4c). The Turnbull stain matched the XFI in three lesions while in the fourth it did not demonstrate the iron ring (Fig. 2i, l) visible on XFI (Fig. 2h, k-white asterisks). Neither the XFI (Fig. 2b) nor the Turnbull stain (Fig. 2f) showed an iron-containing rim of activated microglia/macrophages in the remaining six smoldering plaques. No zinc was present in the rim (Fig. 2c, j, k). Quantitatively, there was no significant difference in iron (*p* = 0.21) or zinc (*p* = 0.08) between the rims and inactive centers of smoldering lesions.

The completely demyelinated inactive center (Fig. 2a) was devoid of iron in six smoldering lesions, while four lesions (three with and one without an iron ring, all from patients younger than 50 years of age) contained patchy subregions of iron accumulation and iron loss visible on XFI (Figs. 2b, h, k, 4a, b), and iron histochemistry (Fig. 2f, i). These iron-rich regions located within the inactive demyelinated center co-localized with areas of reactive astrogliosis and glial scaring (Fig. 2e). Iron within these patches was significantly increased when compared to the iron in the rest of the demyelinated center (*p* = 0.02), and there was a trend of increase relative to the rim iron (*p* = 0.06), but not compared to the periplaque WM iron (*p* = 0.55). Zinc was low in the inactive demyelinated center of smoldering lesions (Fig. 2c), except in one case where zinc was increased periventricularly (Fig. 2j).

XFI showed that in 8/10 smoldering plaques, iron in the periplaque WM adjacent to the smoldering rim was decreased compared to the remote periplaque WM iron (Figs. 2b, 4c). Three of these eight lesions had a periplaque WM band of very low iron content (Fig. 2k-black asterisks, and the corresponding region in Fig. 2h) located immediately adjacent to the smoldering rim (Fig. 2h, k-white asterisks). Iron histochemistry corresponded with XFI in 5/10 smoldering lesions (Fig. 2f), but failed to identify the periplaque WM iron decrease and the rim-adjacent low iron band (Fig. 2i, l) in 5/10 lesions. Zinc was preserved in all rim-adjacent periplaque WM regions (Fig. 2j, k), except one (Fig. 2c), and was abundant in the remote periplaque WM (Fig. 2c, j, k).

**Fig. 3:**
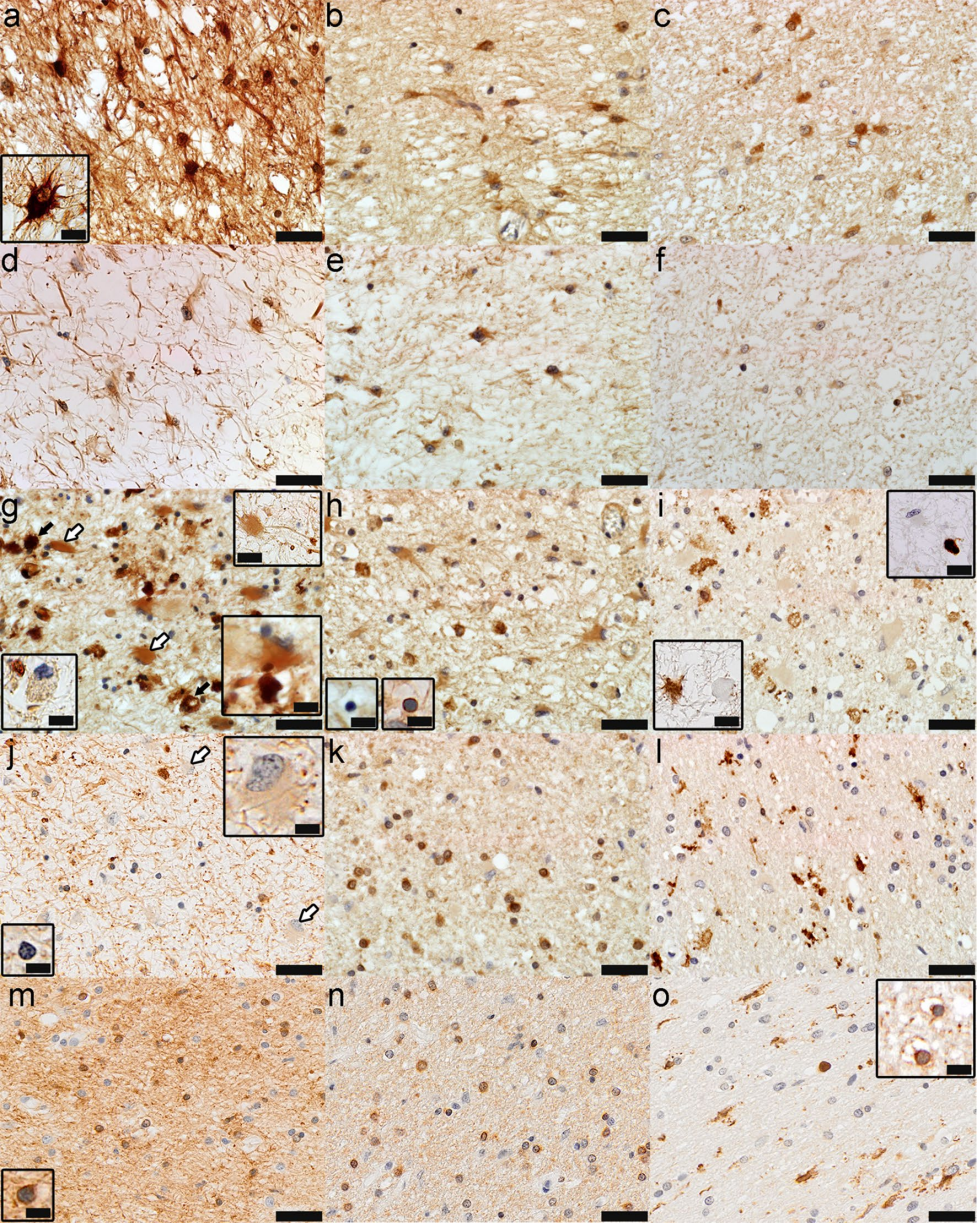
The different subregions of smoldering plaques as defined by their iron content and metalloprotein expression (Case 4 in Fig. 1a). **a**–**c** Iron-rich areas of the inactive center: Fe accumulates in astrocytes (**a**, Fe histochemistry) that express H-ferritin (**b**, FTH) and L-ferritin (**c**, FTL); **d**–**f** Iron-poor areas of the inactive center: astrocytes still accumulate iron but to a lesser extent (**d**, Fe histochemistry), express abundantly H-ferritin (**e**, FTH) but little L-ferritin (**f**, FTL); **g**–**i** The smoldering rim: iron accumulates in dystrophic macrophages (*black arrows, lower right inset*) and reactive astrocytes (*white arrows, lower and upper right insets*), but not all macrophages accumulate Fe (*lower left inset*) (**g**, Fe histochemistry); dystrophic macrophages and reactive astrocytes are immunoreactive for H-ferritin (*insets* show iron-reactive and iron-negative apoptotic oligodendrocytes) (**h**, FTH); dystrophic macrophages, but not reactive astrocytes are immunoreactive for L-ferritin (*lower left inset* shows that reactive astrocytes, but not dystrophic macrophages are immunoreactive for GFAP, while the upper right inset shows that dystrophic macrophages, but not reactive astrocytes are immunoreactive for CD68) (**i**, FTL; *lower left inset*, GFAP; *upper right inset*, CD68); **j**–**l** Rim-adjacent periplaque WM: iron is present in myelinated axons, but not oligodendrocytes (*lower left inset*) or reactive astrocytes (*arrows and upper right inset*) (**j**, Fe histochemistry); oligodendrocytes are immunoreactive for H-ferritin (**k**, FTH) and microglia for L-ferritin (**l**, FTL); **m**–**o** Remote periplaque WM: oligodendrocytes (*inset*) and myelin stain intensely for iron (**m**, Fe histochemistry); oligodendrocytes are immunoreactive for H-ferritin (**n**, FTH) and L-ferritin (*inset* in **o**, FTL), and microglia for L-ferritin (**o**, FTL); *Scale bar* 100 μm; *Inset scale bar* 25 μm

Based on XFI, iron histochemistry and immunohistochemistry for H-ferritin (FTH) and L-ferritin (FTL), we identified five subregions of smoldering plaques:

1. *High iron inactive center regions* Iron-rich areas within the inactive center of smoldering lesions corresponded to iron accumulation within reactive astrocytes and astrocytic fibers (Figs. 3a, 4a) that abundantly expressed FTH (Fig. 3b) and FTL (Fig. 3c).

**Fig. 4:**
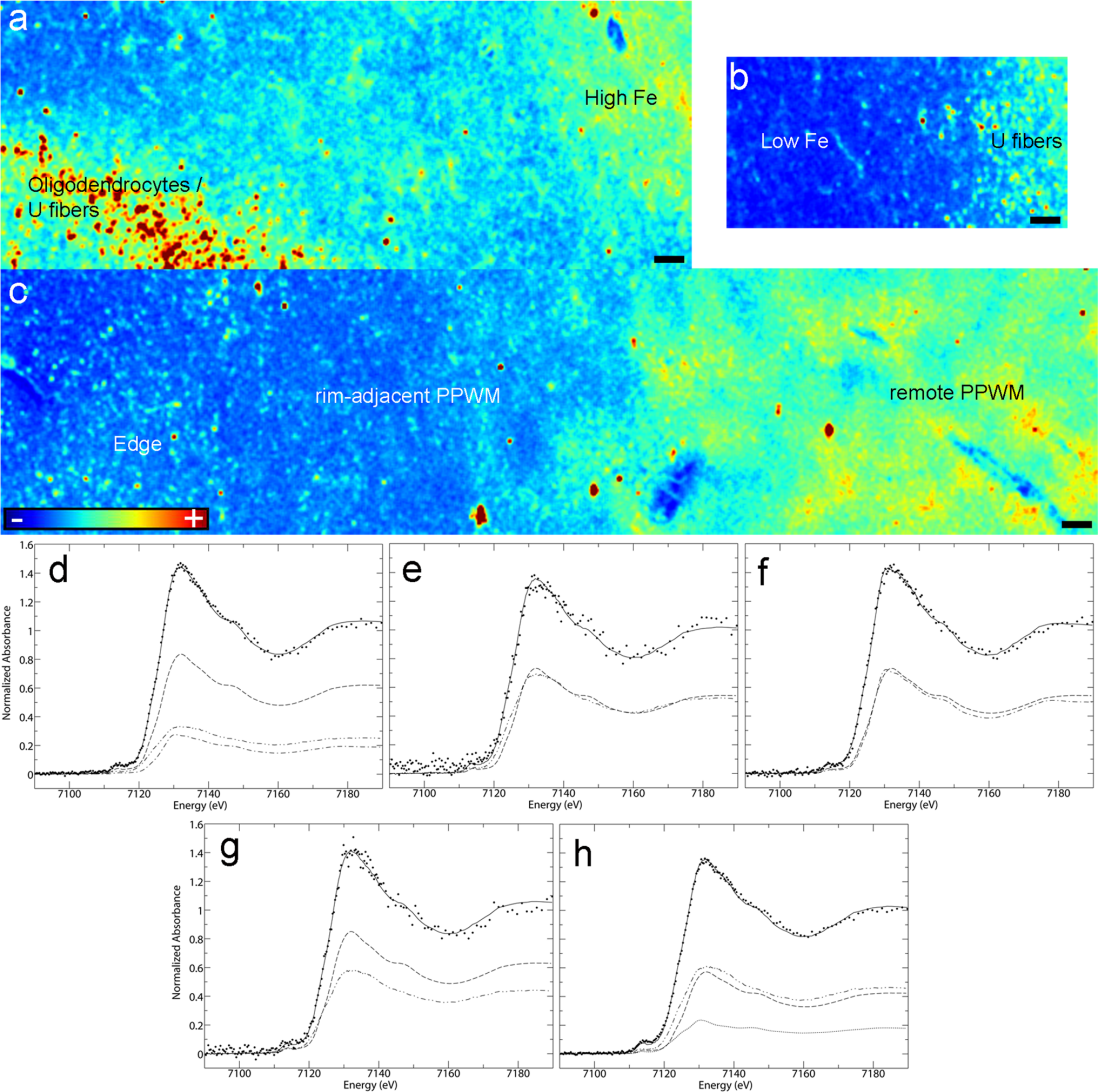
Microprobe XFI and XANES analysis of smoldering lesions (Case 4 in Fig. 1a): **a**–**c** Iron distribution in the various subregions of smoldering plaques (XFI); **d**–**h** Quantitative analysis of Fe K-edge spectra. *Each panel* shows the normalized spectrum of brain tissue (*spaced dotted lines*) together with the best fit (*continuous line*). The fit components are scaled by their relative contributions: components are category as in Table 2. Each category has a unique line type: ferrihydrite (*dashed line*), goethite (*single dotted dashed line*), magnetite (*closed dotted lines*), heme (*double dotted dashed line*). See Table 2 for numerical results of the fits: **d** Iron-rich subregion of the inactive center; **e** Iron-poor subregion of the inactive center; **f** Smoldering rim; **g** Rim-adjacent periplaque WM; **h** Remote periplaque WM. *Scale bar* 90 μm; *Color scales* **a**–**c** represent the normalized total Kα fluorescence counts, proportional to total metal present, from *blue* (lowest) to *red* (highest)
2. *Low iron inactive center regions* Regions of tissue rarefaction located within the high iron patches, and between these and the smoldering rims or the periplaque WM, contained sparse small reactive astrocytes and thin glial fibers that contained low amounts of iron (Figs. 3d, 4b) which expressed abundant FTH (Fig. 3e) but little FTL (Fig. 3f).
3. *Smoldering rim* Iron was present in reactive astrocytes (white arrows in Figs. 3g, 4c) and cells resembling macrophages (black arrows in Figs. 3g, 4c). These cells stained intensely for iron (Fig. 3g, g lower right inset), were CD68-immunopositive (Fig. 3i upper right inset), GFAP-immunonegative (Fig. 3i lower left inset), and dystrophic (Fig. 3g, i insets) [22]. Normal appearing macrophages staining faintly for iron was also present (Fig. 3g lower left inset). Reactive astrocytes stained intensely for iron (Fig. 3g, g right insets) and were often in close contact with, and appeared to incorporate iron from macrophage remnants (Fig. 3g right upper and lower insets). Reactive astrocytes, macrophages, and their remnants were immunoreactive for FTH (Fig. 3h), but only the latter were immunoreactive for FTL (Fig. 3i). Most oligodendrocytes present in the smoldering rim showed pyknotic nuclei consistent with apoptosis (Fig. 3h insets), did not stain for iron (Fig. 3h lower left inset), were faintly immunoreactive for FTH (Fig. 3h) but immunonegative for FTL (Fig. 3i). Iron-loaded apoptotic oligodendrocytes were occasionally observed (Fig. 3h lower right inset).
4. *Rim-adjacent periplaque white matter* The rim-adjacent periplaque WM contained oligodendrocytes (Fig. 3j, j lower left inset) and reactive astrocytes (Fig. 3j arrows and upper right inset) that did not stain for iron. High resolution XFI showed that iron was present in cells in this region (Fig. 4c). Myelin and few macrophages/microglia in the smoldering rim-adjacent periplaque WM stained for iron (Fig. 3j). Oligodendrocytes were immunoreactive for FTH (Fig. 3k). Only microglia were immunoreactive for FTL (Fig. 3l).
5. *Remote periplaque white matter* The remote periplaque WM showed the normal iron patchy appearance and the U fiber high iron content (Figs. 2b, f, h, i, l, 4a–c). Most oligodendrocytes (Fig. 3m, m inset) and myelin (Fig. 3m) stained intensely for iron. Oligodendrocytes were immunoreactive for FTH (Fig. 3n) and FTL (Fig. 3o inset), whereas microglia were immunoreactive for FTL (Fig. 3o).

#### XANES analysis across smoldering plaque subregions

Iron K near edge spectra (Suppl. Fig. 2a–c) from these five subregions were compared in Fig. 4d–h and Table 2, and showed that the highly ordered ferrihydrite of ferritin predominated in most samples, except in the high iron areas of the smoldering plaque inactive center and the smoldering rim, where goethite accounted for about one third (30%) and one half (50%), respectively, of all ferric oxyhydroxides. Magnetite accounted for 17% of the total iron (30% of ferric oxides) present in the remote periplaque WM. Heme iron represented an important component in the remote periplaque WM (44%), rim-adjacent periplaque WM (42%) and low iron inactive center areas (50%), but its proportion decreased dramatically in the smoldering rim (0%) and high iron areas (24%).

**Table 2:** Percentage contributions ^a^ of spectra of iron compounds and metalloproteins

Lesion	Ferric oxide			Heme	Fit-error^b^
	Ferrihydrite	Goethite	Magnetite		
Smoldering					
1. High iron inactive center	57 (1.4)	19 (1.2)		24 (0.6)	0.236
2. Low iron inactive center	50 (1.8)			50 (1.8)	0.239
3. Smoldering rim	50 (6)	50 (5)			0.447
4. Periplaque WM (rim adjacent)	58 (1.3)			42 (1.3)	0.123
5. Periplaque WM (remote)	39 (6)		17 (7)	44 (10)	0.217
Chronic inactive					
1. High iron	88 (9)			12 (9)	0.828
2. Low iron	79 (15)			21 (15)	0.179

^a^Percentage contribution of iron species to total iron. Precisions, shown in parenthesis, are calculated as three times the estimated standard deviation, as derived from the diagonal elements of the covariance matrix; best fit using all model spectra in Suppl. Table 3

^b^Fit error (×10^−3^) or residual is the average of the sum of the squares of the differences between the observed and calculated signals for all data points in the spectrum

#### Chronic inactive white matter plaques

**Fig. 5:**
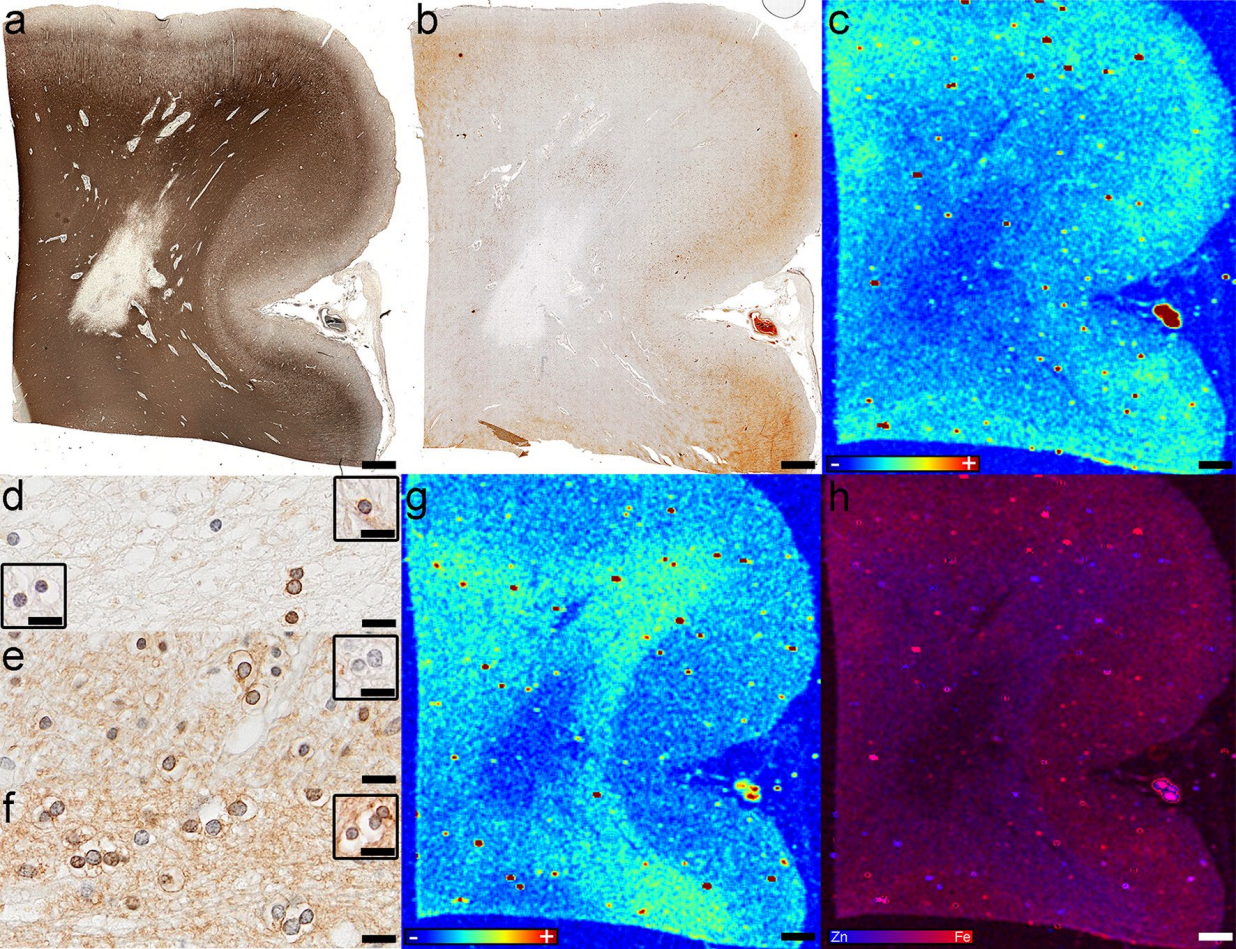
Iron and zinc in inactive lesions; Case 13 in Fig. 1a: **a** The demyelinated lesion is seen as the lack of myelin immunoreactivity (PLP); **b** Iron seems to be lost in the lesion and most periplaque WM (Fe histochemistry); **c** XFI shows that iron is lost in the demyelinated lesion, but gradually decreases in the periplaque WM from the normal appearing WM toward the lesion (XFI); **d** Oligodendrocytes are still present in lesions: some are immunoreactive for H-ferritin (FTH), and both L-ferritin-immunopositive (*upper right inset*; FTL) and L-ferritin-immunonegative (*lower left inset*; FTL) oligodendrocytes are observed; **e** Oligodendrocytes and myelinated axons are immunoreactive for H-ferritin (FTH) but not L-ferritin (*inset*, FTL) in the periplaque WM; **f** Oligodendrocytes and myelinated axons are immunoreactive for H-ferritin (FTH) and L-ferritin (*inset*; FTL) in the normal appearing WM; **g** Zn is lost in the demyelinated lesion (XFI); **h** Fe and Zn are both lost in inactive demyelinated lesions, while iron is decreased in the normal appearing WM (XFI). *Scale bars* **a**–**c**, **g**, **h** 3 mm; *Scale bars* **d**–**f** 50 μm; *Color scales* **c**, **g** represent the normalized total Kα fluorescence counts, proportional to total metal present, from *blue* (lowest) to *red* (highest); *Color scale* **h** represents the overlay of the normalized total Fe and Zn Kα fluorescence counts, proportional to total metal present, from *blue* (Zn) to *red* (Fe)

**Fig. 6:**
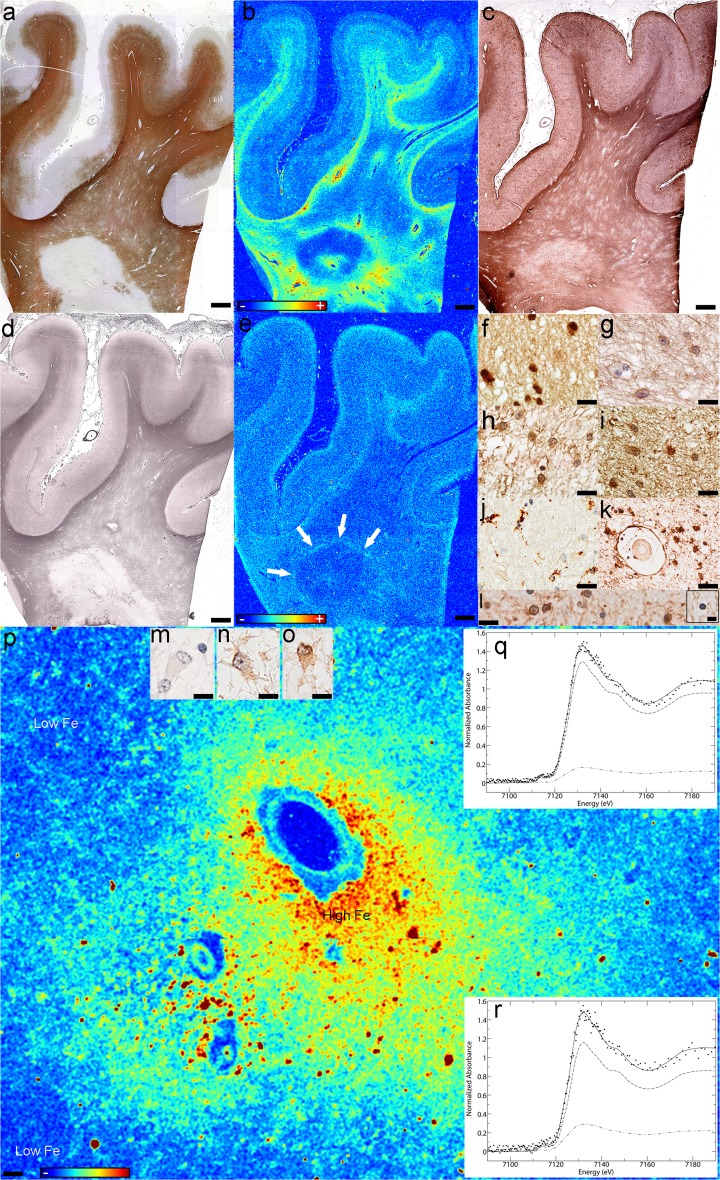
Iron and zinc in iron accumulating inactive lesions. Case 11 in Fig. 1a: **a** The demyelinated lesion is seen as the lack of immunoreactivity for myelin (PLP); **b** Fe accumulates in a concentric pattern in the demyelinated lesion (XFI), and **c** co-localizes with areas of reactive astrocytosis (GFAP), but **d** not with axonal preservation (silver impregnation); **e** Zn is lost in most of the lesion, periplaque and normal appearing WM, except a region (arows) at the lesion’s edge (XFI); Reactive astrocytes **f** accumulate iron (Fe histochemistry), and are immunoreactive for: **g** GFAP (GFAP), **h** H-ferritin (FTH) and **i** L-ferritin (FTL). **j** Activated microglia are present within the iron-rich regions of the lesion but do not stain for iron (CD68); **k** Perivascular astrocytes and the glia limitans stain for iron in these iron rich areas (Fe histochemistry); **l** Both iron-positive and iron-negative (inset) oligodendrocytes are present in the normal appearing WM (Fe histochemistry); Astrocytes in the low-iron subregions of the inactive demyelinated lesions are **m** negative for iron (Fe histochemistry), **n** but immunoreactive for H-ferritin (FTH) and **o** L-ferritin (FTL); **p** Iron accumulates perivascularly in astrocytes (XFI); **q**, **r** Quantitative analysis of Fe K-edge spectra. Each panel shows the normalized spectrum of brain tissue (*spaced dotted lines*) together with the best fit (*continuous line*). The fit components are scaled by their relative contributions: components are category as in Table 2. Each category has a unique line type: ferrihydrite (*dashed line*), heme (*double dotted dashed line*). See Table 2 for numerical results of the fits: **q** Quantitative analysis of Fe K-edge spectra in iron rich regions; **r** Quantitative analysis of Fe K-edge spectra in iron-poor regions; *Scale bars* **a**–**e** 3 mm; *Scale bars* **f**–**l** 50 μm; *Scale bars* **l** *inset*, **m**–**o** 12.5 μm; *Scale bar* **p** 90 μm; *Color scales* **b**, **e**, **p** represent the normalized total Kα fluorescence counts, proportional to total metal present, from *blue* (lowest) to *red* (highest)

We analyzed nineteen inactive demyelinated plaques (Table 1; Figs. 5a, 6a, Suppl. Fig. 1a, b, Suppl. Fig. 5a) from 11 tissue blocks of ten MS patients (six with SPMS, one with and five without attacks; one with PPMS; three with uncertain course). Eighteen inactive plaques (95%) showed iron loss on XFI (Fig. 5c, h, Suppl. Fig. 1c, Suppl. Fig. 5b, d) and iron histochemistry (Fig. 5b, Suppl. Fig. 5e). XFI showed loss of zinc in inactive lesions (Fig. 5g, h, Suppl. Fig. 1d, Suppl. Fig. 5c, d). While iron staining showed iron loss in the periplaque WM (Fig. 5b, Suppl. Fig. 5e), XFI revealed that periplaque WM iron gradually decreased from the normal appearing WM toward the demyelinated lesion (Fig. 5c, Suppl. Fig. 1c, Suppl. Fig. 5b). The periplaque WM zinc distribution was complex, with loss of zinc limited to demyelinated areas in some lesions (Fig. 5g, h; Suppl. Fig. 1d), but extending to the periplaque WM in others (Suppl. Fig. 5c, d). A single inactive lesion revealed a ring of increased zinc around the plaque periphery (Suppl. Fig. 5c, d) without an obvious pathological correlate. Oligodendrocytes were present in the inactive lesions (Fig. 5d) and periplaque WM (Fig. 5e), were FTH-immunoreactive, but their numbers decreased compared to normal appearing WM (Fig. 5f). In the inactive demyelinated lesions both FTL-immunopositive (Fig. 5d upper right inset) and FTL-immunonegative (Fig. 5d lower left inset) oligodendrocytes were present. In periplaque WM, most oligodendrocytes were immunonegative for FTL (Fig. 5e inset), while most oligodendrocytes in the normal appearing WM were immunopositive for FTL (Fig. 5f inset).

A single inactive white matter plaque (Fig. 6a) showed iron increase on XFI and iron histochemistry in a concentric pattern (Fig. 6b). Zinc also had a concentric distribution (Fig. 6e). Iron loss areas separated the rings of iron accumulation (Fig. 6b). The iron rich regions correlated with GFAP (Fig. 6c), but not myelin immunohistochemistry (Fig. 6a) or axonal staining (Fig. 6d). Parenchymal and perivascular reactive astrocytes (Fig. 6g) accumulated iron (Fig. 6f, k, p), and expressed both FTH (Fig. 6h) and FTL (Fig. 6i). Iron negative microglia were present (Fig. 6j). Reactive astrocytes within the lesion’s low iron ring were mildly immunoreactive for FTH (Fig. 6n) and FTL (Fig. 6o), but did not stain for iron (Fig. 6m). Iron was present in the periplaque and normal appearing WM in oligodendrocytes and myelin (Fig. 6l). The myelin had a patchy appearance with alternating areas of normal and pale myelin (Fig. 6a). Apoptotic oligodendrocytes were frequent but did not stain for iron (inset in Fig. 6l).

Similar to the smoldering plaques’ inactive centers, the heme iron decreased between the low (21%) and high iron (12%) subregions with a concomitant increase in ferric iron (79% in low and 88% in high iron subregions), consisting entirely of ferritin ferrihydrite (Suppl. Fig. 2d, Fig. 6q, r; Table 2).

#### Shadow plaques

**Fig. 7:**
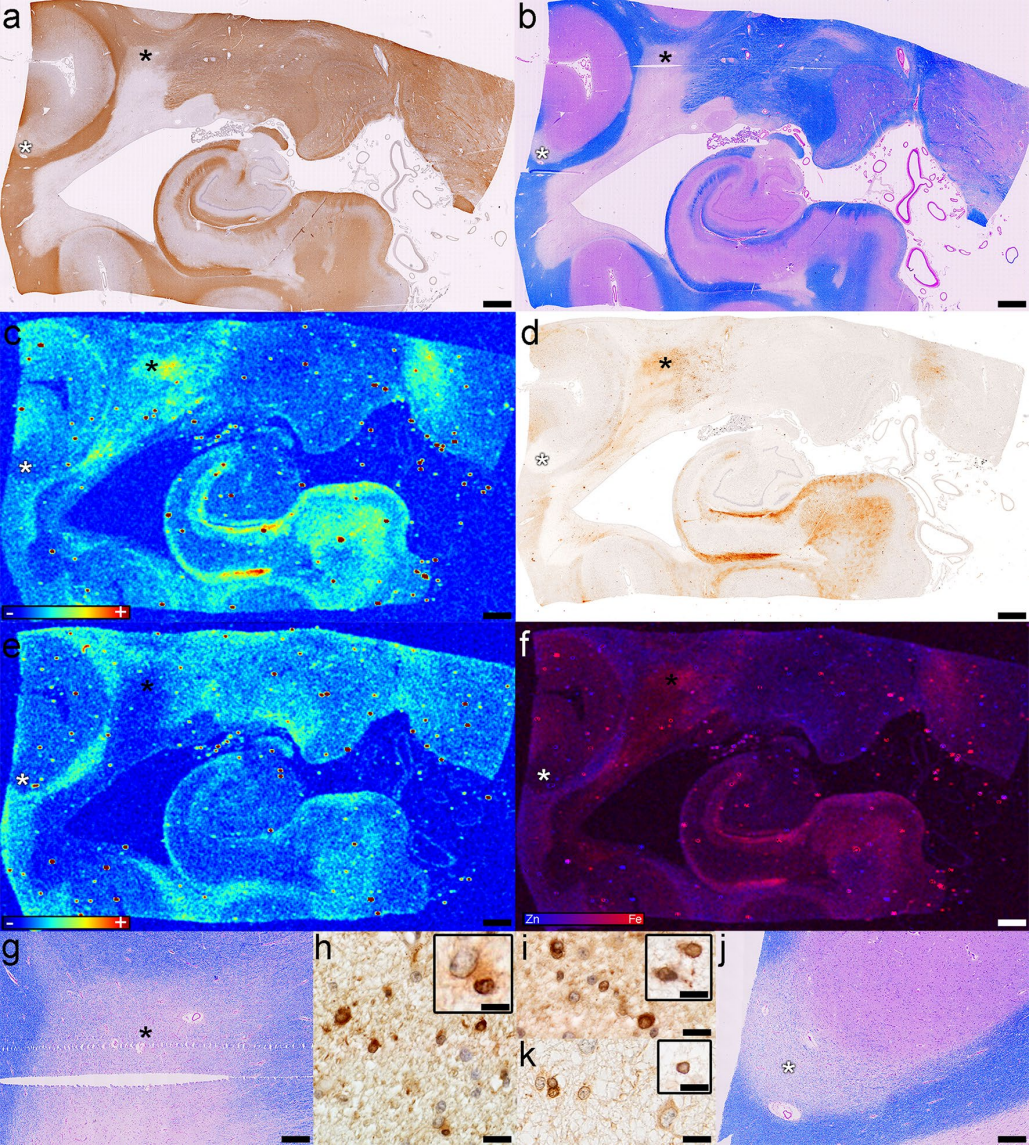
Iron and zinc in shadow plaques (case 14 in Fig. 1a). The shadow plaques (*asterisks*) are seen as **a** milder immunoreactivity for myelin proteins (PLP), and **b** pale Luxol fast blue staining (LFB/PAS); **c** Fe accumulates in one (*black asterisk* in **a** and **b**) of the two shadow plaques, but not the other one (white asterisk in **a** and **b**)  (XFI); **d** The modified Turnbull stain shows a similar iron distribution (Fe histochemistry); **e** Zn is lost in the demyelinated lesion and shadow plaques (XFI); **f** The overlay of Fe and Zn shows the Fe accumulation in one of the two shadow plaques (XFI); **g** Higher magnification of the iron-accumulating (*black asterisk* in **a** and **b**) shadow plaque (Lfb/PAS); Oligodendrocytes with large nuclei are **h** positive for iron (Fe histochemistry), and **i** immunoreactive for H-ferritin (FTH) and L-ferritin (*inset*; FTL); **j** Higher magnification of the iron-poor (*white asterisk* in **a** and **b**) shadow plaque (Lfb/PAS); **k** Oligodendrocytes with normal sized nuclei in the iron-poor remyelinated lesion are immunoreactive for H ferritin (FTH) and L ferritin (*inset*; FTL); *Scale bars* **a**–**f** 3 mm; *Scale bars* **g**, **j** 500 μm; *Scale bars* **h**, **i**, **k** 20 μm; *Color scales* **c**, **e** represent the normalized total Kα fluorescence counts, proportional to total metal present, from *blue* (lowest) to *red* (highest); *Color scale* **f** represents the overlay of the normalized total Fe and Zn Kα fluorescence counts, proportional to total metal present, from *blue* (Zn) to *red* (Fe)

We found five shadow plaques (Table 1; Fig. 7a, b, g, j asterisks) in four tissue blocks from four MS patients (two SPMS, one with and one without attacks; one RRMS; one with uncertain course). The iron distribution was heterogenous among shadow plaques (even those in the same tissue section), with some showing increased iron (Fig. 7c, d, f). Low amounts of zinc were present in remyelinated plaques (Fig. 7e, f). Shadow plaque iron increase was identified by XFI and Turnbull stain (Fig. 7c, d, f). The cells staining most robustly for iron were oligodendrocytes with larger nuclei, a morphological feature consistent with immature oligodendrocytes (Fig. 7h), which stained intensely for FTH (Fig. 7i) and FTL (Fig. 7i inset). Reactive astrocytes were present in iron-rich shadow plaques and some stained for iron (Fig. 7h inset). Mature oligodendrocytes were present in iron-poor remyelinated lesions and were immunoreactive for FTH (Fig. 7k) and FTL (Fig. 7k inset).

#### Cortical demyelinated plaques

We identified 25 cortical plaques (20 subpial—Suppl. Fig. 6a; 2 intracortical—Suppl. Fig. 6i; 3 leukocortical—Suppl. Fig. 6 l) in ten patients (four SPMS, one with and three without attacks; 1PPMS; 2RRMS; three with uncertain clinical course). Iron in cortical lesions had a tendency to be lower than normal appearing cortex (Fig. 1a, b, Suppl. Fig. 6b, c, j, m) as previously reported [44, 71]. Iron histochemistry showed almost no iron in cortical lesions (Suppl. Fig. 6c) with only few iron positive microglia (Suppl. Fig. 6e). However, on XFI iron was decreased but not absent (Suppl. Fig. 6b, h, j, m). Oligodendrocytes (Suppl. Fig. 6 g) and cortical astrocytes (Suppl. Fig. 6 g inset) were mildly immunoreactive for FTH. In the normal appearing cortex, oligodendrocytes, myelin, and few microglia stained for iron (Suppl. Fig. 6d), while only myelin and oligodendrocytes were immunoreactive for FTH (Suppl. Fig. 6f). Microglia were immunoreactive for FTL in lesions and normal appearing cortex. Zinc was present in cortical lesions (Suppl. Fig. 6 k, n). One leukocortical lesion (case 14 in Fig. 1a) had abundant corpora amylacea (Suppl. Fig. 6o) explaining its high zinc content (Suppl. Fig. 6n).

## Discussion

Our study describes distinct and heterogenous patterns of iron and zinc across different chronic MS plaque types. With few remarkable exceptions these metals do not accumulate in chronic MS lesions. Using cutting edge synchrotron techniques that are sensitive and specific to detect metals [52], we report for the first time that astrocytes in large astrogliotic regions in a subset of smoldering and inactive plaques accumulate iron, and safely store it as ferrihydrite in ferritin. Furthermore, we provide preliminary insights into the complex and dynamic relationship of cell-specific iron loading and release and its impact on injury and repair over the chronic MS course.

Smoldering plaques represent the most notable example of iron accumulation within MS plaques. As smoldering plaques are only found among progressive MS patients [19], they are an important plaque type to better understand. Iron has been reported to accumulate in the microglia/macrophages, forming the smoldering rim [1, 22, 39] of these plaques. We, however, find that not all smoldering lesions contain iron-rich microglia/macrophage rims. This may relate to varying degrees of smoldering activity at the plaque edge. We further show that even when an iron-rich rim is present, its iron content is not higher than the normal appearing WM iron, and is only visible because of the iron-poor rim-adjacent periplaque WM separating the two regions. The distribution of iron differs between these two regions: oligodendrocytes and myelin in normal appearing WM stain most intensely for iron [8, 63], whereas iron in smoldering rims is localized to dystrophic and normal microglia/macrophages [22], as well as to astrocytes.

It has been suggested that the destruction of iron-loaded oligodendrocytes in MS active lesions may release iron extracellularly, where it is subsequently picked up by microglia/macrophages. Degeneration of these iron-loaded microglia/macrophages may induce a second wave of iron release, with subsequent axonal iron accumulation, oxidative damage and neurodegeneration [22]. While it is known that reactive astrocytes incorporate iron [22], we describe for the first time that reactive astrocytes forming large areas of astrogliosis accumulate iron in a subset of MS patients. These iron-rich gliotic patches may represent another potential protective barrier the brain mounts when faced with degeneration of iron-loaded microglia/macrophages. This is further supported by our finding of astrocytes in close contact with, and incorporating iron-reactive fragments from iron-loaded macrophages.

Moreover, we observe the highest iron accumulating reactive astrogliotic regions in inactive centers of smoldering lesions, further suggesting that reactive astrocytes may play an important role in iron uptake and storage in chronic MS lesions. This is not surprising since upon inflammatory activation astrocytes elevate their capacity to incorporate iron [42, 54, 73], and their ability to resist iron-dependent oxidative stress [35]. Immunohistochemistry confirms that the iron-loaded astrocytes within these gliotic patches are in fact immunoreactive for iron storage proteins, consistent with their ability to safely incorporate large amounts of iron in ferritin [16]. Furthermore, XANES analysis, although limited to two cases, indicates at least half of the iron in both the smoldering rims and high-iron gliotic regions within the inactive centers of these plaques consists of the highly ordered ferrihydrite of ferritin. Surprisingly, a high proportion of this accumulated iron represents goethite, the predominant iron bio-mineralization component of haemosiderin in thalassaemia [66]. While the mechanism of goethite formation is undefined, it is unlikely that it forms from ferritin’s ferrihydrite [10, 11]. Iron in goethite is less soluble and released less readily than iron of ferritin [2, 40, 67]. Because XFI cannot distinguish between cell types, it is conceivable that astrocytes pick up the goethite already biomineralized by macrophages, or they biomineralize the iron picked up from macrophages. In either case an important proportion of iron is stored as goethite that is even less bioavailable and less toxic to cells than iron of ferritin.

The fate of iron-loaded astrocytes in MS lesions is unknown. Astrocytes resist iron overload until their antioxidant potential is exhausted [35]. It is possible that while astrocytic iron accumulation is protective in the short term, incorporating increasing amounts of free iron over time as a consequence of continuous inflammatory activity with ongoing destruction of iron-loaded oligodendrocytes and macrophages will eventually exhaust the astrocytes’ antioxidant defenses, thereby leading to their death. This may explain the presence of tissue rarefaction observed surrounding high iron astrocytic patches within the inactive center of smoldering plaques. While iron is decreased in these tissue-rarefied regions, its chemistry more closely resembles that of the periplaque WM with half sheltered in ferritin and half associated with heme proteins. The latter may be hemoproteins that are involved in mitochondrial respiration, suggesting a potential metabolic reversal of astrocytes from an iron-storage to a normal phenotype. Alternatively, the heme observed in rarefied gliotic low iron regions may consist of brain globins, which are neuroprotective heme-containing proteins [5, 53]. Since XANES analysis only interrogates the heme iron, further studies will need to elucidate their exact nature, and contribution.

Iron efflux from astrocytes is known to be important for remyelination [60]. The iron-induced oxidative destruction of iron-loaded astrocytes in a noninflammatory setting could provide a double benefit: the removal of the glial scar that impairs the migration of oligodendrocyte precursor cells into lesions [25], and the release of iron crucial for efficient remyelination [63]. Alternatively, astrocytes can provide iron to oligodendrocytes through the ferroportin-ceruloplasmin system [22, 41, 70]. However, in the setting of even a minimal inflammatory milieu, as expected in long standing chronic MS [18], iron liberation from astrocytes would serve to augment oxidative damage rather than promote remyelination.

Age and disease duration may contribute to the development of iron-rich rims or iron-rich gliotic patches observed in a subset of smoldering lesions. All but one of the smoldering lesions with an iron rim and all smoldering plaques with iron-rich cores were observed in younger patients (<50 years old) with less than 15 years disease duration. It is possible that younger patients during earlier disease stages have more active inflammatory disease resulting in more pronounced oligodendrocyte destruction with subsequent iron release compared to older longstanding patients with less active disease [18]. Alternatively, the variable presence of iron rims may reflect differences in macrophage polarization [55] or changes in macrophage polarization as a result of iron loading [39].

While the majority of chronic inactive lesions showed iron loss, one chronic plaque from an elderly patient [62] demonstrated concentric rings of iron increase and loss. Iron concentration within this lesion resembled the inactive iron-rich gliotic centers observed among smoldering plaques with an accumulation of iron within reactive astrocytes immunoreactive for both FTH and FTL, higher ferrihydrite concentrations in iron-rich versus iron-poor rings, and a concomitant decrease in heme iron. These observations suggest that in some chronic lesions iron safely accumulates in ferritin in astrocytes, but not microglia.

Interestingly, in the periplaque WM of most smoldering and chronic inactive plaques, there is a gradient of iron loss towards the lesion edge, with three smoldering plaques demonstrating a clear ring of iron loss adjacent to the smoldering rim. Iron in the periplaque WM is significantly lower than iron in the normal appearing WM. While iron histochemistry shows that iron-positive myelinated axons are present in the periplaque WM, FTH-immunoreactive oligodendrocytes in the periplaque WM do not stain for iron. This suggests that oligodendrocytes in the periplaque WM are able to dispose of their iron, a finding compatible with previous studies reporting active iron export from periplaque WM oligodendrocytes [22]. Iron-loaded oligodendrocytes are more vulnerable to cytokine toxicity than iron-depleted oligodendrocytes [73]. Therefore, this disposal of iron by periplaque WM oligodendrocytes may be a protective, but transient, mechanism oligodendrocytes employ when faced with the advancing front of inflammation [73]. Microglia may pick up the oligodendrocyte-released iron, and upon activation and in the context of iron loading, increase their release of proinflammatory cytokines, thereby switching from a trophic to a toxic phenotype [74]. The presence of iron-negative reactive astrocytes in the periplaque WM indicates that CNS may be prepared to deal with the potential iron release that ensues.

We have also observed iron-negative FTH immunoreactive oligodendrocytes within inactive demyelinated white matter and cortical lesions. While their number is decreased compared to the periplaque and normal appearing WM, these oligodendrocytes are characterized by normal morphology, suggesting they may be capable of surviving in lesions despite the absence of iron.

A lack of iron within oligodendrocytes may impair their remyelinating capacity [61, 63]. Previous studies have suggested that iron does not accumulate in remyelinated plaques [22], and indeed iron was decreased in remyelinated lesions when analyzed as a group. However, when examined individually, we found a heterogeneous concentration of iron with some remyelinated plaques displaying increased iron. Oligodendrocytes within these shadow plaques stained robustly for iron, and were characterized by larger nuclei, compatible with an immature phenotype [30]. At the other end of the spectrum, we observed remyelinated lesions where oligodendrocytes with normal morphology did not stain for iron. These observed differences may be explained by differences between early versus late remyelination. When remyelination is ongoing, an increased amount of iron is required, whereas when remyelination is complete, the iron content may decrease to levels at or below those of the normal appearing WM [61, 63].

In addition to iron, zinc is important in the structure and compaction of myelin [14, 29, 64]. Zinc is decreased in most lesions and its distribution parallels that of myelin. Demyelinated white matter lesions are devoid of zinc, and zinc decreases in the periplaque WM showing myelin pallor. Only three white matter lesions had a moderate zinc increase, but their pathologic correlate remains unknown. A single smoldering plaque showed increased periventricular zinc, and two inactive lesions were characterized by rings of zinc at their borders. Cortical lesions showed reduced, but not absent, zinc, likely due to the fact that most cortical zinc is concentrated in neurons [17]. Zinc was increased in one leukocortical lesion where it localized to corpora amylacea [65].

We also analyzed the relationship between iron and zinc with age and MS disease duration. As part of normal aging, iron accumulates in microglia and astrocytes [9], presumably due to a cytokine-induced phenomenon [13]. In MS, due to the inflammatory environment [18], iron accumulation is likely accelerated and present already at disease onset. It has been reported that iron decreases in the normal appearing WM of MS patients with increasing disease duration, presumably due to the destruction of iron-loaded oligodendrocytes [22]. We observed that iron content also decreases within MS plaques with increasing age. In contrast, neither disease duration, nor age was related to zinc content within MS plaques.

Whether there is a role for iron chelation in MS remains controversial [12, 61, 69] and is based on the premise that iron accumulates in lesions. We have generally observed the opposite, as chronic MS plaques tended to be deficient in iron. Chelation of these minimal amounts of iron may suppress oligodendrocyte metabolic activity and induce cell death [73]. Chelation of iron from remyelinating plaques could also be detrimental, given the potentially trophic effect of iron and ferritin on oligodendrocytes and myelination [58, 74]. However, oligodendrocytes are still functional in normal white matter [9], and survive in chronic and remyelinated lesions, as well as periplaque WM despite showing no stainable iron. Since iron chelation may not negatively affect all oligodendrocytes, it may be considered for the smoldering and chronic MS lesions where iron accumulates in astrocytes, macrophages or microglia. Removing the iron from iron-filled astrocytes could potentially increase their ability to accumulate more iron, thereby prolonging their survival. Iron removal from iron-rich activated macrophages and microglia in smoldering rims may also decrease the release of inflammatory cytokines and protect oligodendrocytes [74]. Further studies will need to establish if there is a role for iron chelation in MS given its potential detrimental and beneficial effects.

The relationship between iron metabolism diseases and MS is currently unknown. Although genetic hemochromatosis does not increase MS severity [57], a recently developed hemochromatosis mouse model indicates that brain iron accumulation alters the myelin-related transcriptome [23, 24]. While this relationship may be elucidated by EAE induction in this model, the relevance to MS remains to be determined.

Our study highlights the limitations of iron histochemistry [38]. Although iron staining approximates well the XFI iron in iron-rich areas, it grossly underestimates iron in iron-poor lesions, normal appearing WM and periplaque WM. This is explained by the XFI’s high sensitivity and specificity for iron [52], and by XANES showing that iron-rich areas mostly contain ferric non-heme iron, while iron-poor regions contain more heme iron. Another limitation which impacts both iron histochemistry and XFI is the use of formalin-fixed paraffin-embedded tissue. Formalin fixation is known to leach metals from tissues, although the extent of leaching is debatable [6, 7, 59]. Paraffin embedding may also accentuate this phenomenon, and further oxidize metals, possibly accounting for why no ferrous iron was detected [21]. The limited number of cases from which XANES spectra have been collected is another study limitation. Nevertheless, these data are valuable and complementary to ferritin immunohistochemistry, with both approaches indicating that most iron is safely stored in ferritin. Further studies need to establish whether our findings are broadly applicable to iron accumulation in a larger cohort of smoldering and chronic plaques.

## Conclusion

We describe novel findings with respect to the localization of iron and zinc in MS chronic lesions, and provide insights into the role of metals in MS lesion evolution and repair. Our findings highlight that iron and zinc homeostasis in MS pathogenesis is more complex and dynamic than previously reported in published pathological-radiographic correlative studies. We observed that while iron is decreased in most chronic MS lesions, there is a subset of smoldering and inactive lesions where reactive astrocytes safely accumulate ferric iron as ferrihydrite. Zinc in MS lesions is generally decreased, paralleling the myelin loss. We also show that not all smoldering rims contain iron and we describe a single chronic inactive lesion where iron accumulates concentrically within the lesion. Further studies are needed to determine whether MRI features reportedly associated with iron rims around smoldering and inactive plaques differ. Although our study focused on chronic non-active MS lesions, XFI-pathological correlative studies examining iron and zinc in actively demyelinating lesions are ongoing, to better define their role in early lesion formation. Understanding the balance and timing of trophic, toxic, and antioxidant effects of biometals in early and chronic MS is critical for developing pharmacological interventions that either facilitate repair or disrupt the chain of events caused by metal-induced oxidative stress and MS tissue injury. XFI-MRI correlative studies will permit development and validation of specific metal detection methods paving the way to novel metal-based biomarkers to monitor disease activity and progression in MS.

## Electronic supplementary material

Below is the link to the electronic supplementary material.

Supplementary material 1 (PDF 137 kb)

Supplementary material 2 (TIFF 3683 kb)

Supplementary material 3 (TIFF 8875 kb)

Supplementary material 4 (TIFF 565 kb)

Supplementary material 5 (TIFF 1106 kb)

Supplementary material 6 (TIFF 6503 kb)

Supplementary material 7 (TIFF 12562 kb)
