# Supplementary material for: Pathogenic implications of distinct patterns of iron and zinc in chronic MS lesions
Source: Acta Neuropathol. 2017 Mar 22;134(1):45–64. doi: 10.1007/s00401-017-1696-8 (PMC5486634; doi:10.1007/s00401-017-1696-8)
Supplement: Supplementary file 1 — Supplementary material 1 (PDF 137 kb) [file 401_2017_1696_MOESM1_ESM.pdf]

Electronic Supplementary Material

Supplementary tables

Supplementary Table 1. Summary of patient clinical characteristics

Characteristic	Summary
Number of patients	18
Gender, n (%)	
Female	10 (56%)
Male	8 (44%)
Age at death (years)	
Median (Q1, Q3)	66 (45, 72)
Range	19 to 82
Disease duration (years)*	
Median (Q1, Q3)	16 (4, 28)
Range	1 to 42
Clinical course, n (%)	
Relapsing remitting MS	2 (11%)
Secondary progressive MS with attacks	4 (22%)
Secondary progressive MS without attacks	6 (33%)
Primary progressive MS	1 (6%)
Monophasic to death within 1 year	1 (6%)
Unavailable	4 (22%)
Regions of interest per block	
Median (Q1, Q3)	7 (5, 9)
Range	3 to 19

Supplementary Table 2. Primary antibodies used for immunocytochemistry

Antibody	Target	Antibody type	Dilution	Source
**PLP**	Myelin proteolipid protein	Rabbit polyclonal	1:500	Serotec, Oxford, USA
**MOG**	Myelin oligodendrocyte glycoprotein	Rabbit monoclonal	1:1000	Abcam, USA
**MAG**	Myelin-associated glycoprotein	Rabbit monoclonal	1:500	Sigma, USA
**CD68**	Macrophages/microglial	Mouse monoclonal	1:1000	Dako, Denmark
**KiM1P**	Macrophages/microglial	Mouse monoclonal	1:1000	Dr. Radzun, University of Göttingen, Germany
**CD3**	All T cells	Rat monoclonal	1:400	Serotec, USA
**CD8**	Cytotoxic T cells	Mouse monoclonal	1:50	Dako, Denmark
**NF**	Phosphorylated neurofilament	Rabbit polyclonal	1:2000	Chemicon, USA
**GFAP**	Glial fibrillary acidic protein	Mouse monoclonal	1:4000	Dako, Denmark)
**FTH**	Heavy chain ferritin	Rabbit polyclonal	1:1000	Abcam, USA
**FTL**	Light chain ferritin	Goat polyclonal	1:300	Abcam, USA

Supplementary Table 3. Iron model compounds and metalloproteins used in fits

Model compounds and metalloproteins[a]	Chemical formula	Coordination
Fe (III)		
1. Ferrihydrite (horse spleen ferritin)	FeOOH	6O
2. Human frataxin holopolymer	FeOOH	6O
3. Goethite	FeOOH	6O
4. Hematite	α-Fe_2O_3	6O
5. Magnetite	$Fe^{3+}{}_2Fe^{2+}O_4$	6O
6. Ferric citrate	$FeC_6H_5O_7$	3O
7. Heme (human met- hemoglobin)		5N
8. Hemin (bovine)		4N, 1Cl
Iron-sulfur cluster		
9. Ferredoxin - oxidized (Anabaena)	$[Fe_2\text{-}S_2(SR)_2]^{2-}$	4S
10. Rubredoxin - oxidized (P. furiosus)	$[Fe(SR)_4]^{1-}$	4S
11. Iron regulatory protein - oxidized	$[Fe_3\text{-}S_4]^{+}$	4S
12. Rubredoxin – reduced (P. furiosus)	$[Fe(SR)_4]^{2-}$	4S
13. Ferredoxin – reduced (Anabaena)	$[Fe_2(SR)_4]^{3-}$	4S
Fe (II)		
14. Ferrous sulfide	FeS	1S
15. Ferrous sulfate	$FeSO_4$	6O

[a]Spectra from the listed compounds are used in the fit to represent a general class or category of compound, rather than of the particular species from which the spectrum was obtained.

Supplementary figure legends

Supplementary Figure 1. Correlation between histology and XRF (case 10 in Fig. 1a). **a** Sections stained for proteolipid protein were scanned at 40x magnification and whole maps saved as .jpeg files (PLP). **b** Regions of interest (ROI) were identified and outlined (PLP). XFI images for **c** Fe (XFI) and **d** Zn (XFI) were generated, and displayed side by side with the histology maps. **e-l** The regions of interest were outlined on the XFI maps using the histology maps as guides (XFI): **e, i** Lesion; **f, j** Periplaque WM; **g, k** Normal appearing WM; **h, l** Normal appearing cortex; **e-h** Fe; **i-l** Zn; *Scale bars* 3 mm; *Color scales* represent the normalized total Kα fluorescence counts, proportional to total metal present, from *blue* (lowest) to *red* (highest).

Supplementary Figure 2. Points of interest selected on the microprobe XFI maps of one smoldering and one inactive lesion from which XANES spectra were collected: **a-c** smoldering lesions (Case 4 in Fig. 1a): **a** black squares indicate the points of interest selected from the high iron inactive center; **b** white circles indicate the points of interest selected from the low iron inactive center; **c** large black squares indicate the points of interest selected from the smoldering rim, black circles indicate the points of interest selected from the rim-adjacent periplaque WM, and the small black squares indicate the points of interest selected from the remote periplaque WM; **d** chronic inactive lesion (Case 11 in Fig. 1a): black circles indicate the points of interest selected from the high iron regions, and the black squares indicate the points of interest selected from the low iron regions; *Scale bars* 90 μm;

Color scales represents the normalized total Kα fluorescence counts, proportional to total metal present, from *blue* (lowest) to *red* (highest).

Supplementary Figure 3. Relationship between metals and age in MS; **a** Scatter plots of iron levels by patient age. Point colors indicate patients' disease duration group. Each scatter plot includes a least-squares regression line for reference. Mixed models indicated significant negative associations across region types; **b** Scatter plot of zinc levels by patient age. Point colors indicate patients' disease duration group. Each scatter plot includes a least-squares regression line for reference. Mixed models indicated no significant evidence of an association between age and zinc.

Supplementary Figure 4. **a** Iron by disease duration with a least squares line added for reference. Point colors indicate the subject's age group; **b** Zinc by disease duration with a least squares line added for reference. Point colors indicate the subject's age group; **c** Distribution of iron and zinc by region type. Point colors indicate the subject's age group.

Supplementary Figure 5. Chronic inactive demyelinated lesion in case 7 in Fig. 1a: **a** The demyelinated lesion is seen as the lack of Lfb stain (LFB/PAS); **b** Iron is lost in the lesion and decreased in the periplaque WM (XFI); **c** Zinc is decreased in the lesion and periplaque WM, but a ring of zinc (white arrows) whose pathological correlate remains unknown is visible at the lesion's edge (XFI); **d** The overlay of iron

and zinc shows the loss of iron and zinc from the lesion and periplaque WM, and the presence of the zinc ring at the lesion's borders (XFI); **e** Unlike XFI, the iron histochemistry shows the complete absence of iron in the lesion, periplaque WM and normal appearing WM (Fe histochemistry); **f** Immunohistochemistry for H-ferritin shows absence of immunoreactivity in the lesion, but preserved immunoreactivity in the periplaque WM and normal appearing WM (FTH); *Scale bars* 3 mm; *Color scales* **b, c** represent the normalized total Kα fluorescence counts, proportional to total metal present, from *blue* (lowest) to *red* (highest); *Color scale* **d** represents the overlay of the normalized total Fe and Zn Kα fluorescence counts, proportional to total metal present, from *blue* (Zn) to *red* (Fe).

Supplementary Figure 6. Fe and Zn in cortical lesions. **a-h** Subpial lesion in case 18 in Fig. 1a: **a** The demyelinated subpial lesion is seen as the lack of immunoreactivity for myelin extending from the pial surface, involving all cortical layers and, in this case, also extending in the subadjacent white matter (PLP); **b** Fe is diminished, but not absent in the subpial lesion (XFI), while **c** Fe seems to be absent on the modified Turnbull stain (Fe histochemistry); **d** Oligodendrocytes stain for Fe in the normal appearing cortex (Fe histochemistry), and **e** microglia stain for Fe in the subpial lesion (Fe histochemistry); **f** Oligodendrocytes are immunoreactive for H-ferritin in normal appearing cortex (FTH); **g** Oligodendrocytes and astrocytes (inset) are immunoreactive for H-ferritin in the subpial lesion (FTH); **h** Microfocused XFI shows that Fe is still present in the subpial lesion (XFI); **i-k** Intracotical lesion in case 6 in Fig. 1a: **i** The demyelinated intracortical lesion is seen as the perivascular

lack of immunoreactivity for myelin (PLP); **j** Fe and **k** Zn are still present in the intracortical lesion (XFI); **l-o** Leukocortical lesion in case 14 in Fig. 1a: **l** The demyelinated leukocortical lesion is seen as the lack of immunoreactivity for myelin extending from the cortex within the subadjacent white matter, and not reaching the pial surphace (PLP); **m** Fe is decreased but still present in the leukocortical lesion (XFI); **n** Zn is increased in this leukocortical lesion (XFI), and **o** seems to be contained within corpora amylacea (LFB/PAS); *Scale bars* **a – c** 1 mm; *Scale bars* **i - n** 500 μm; *Scale bars* **d - g** 40 μm; *Scale bar* **h** 180 μm; *Scale bar* **o** 20 μm; *Color scales* **b, h, j, k, m, n** represent the normalized total Kα fluorescence counts, proportional to total metal present, from *blue* (lowest) to *red* (highest).
