# Supplementary figures and images for: Pathogenic implications of distinct patterns of iron and zinc in chronic MS lesions
Source: Acta Neuropathol. 2017 Mar 22;134(1):45–64. doi: 10.1007/s00401-017-1696-8 (PMC5486634; doi:10.1007/s00401-017-1696-8)

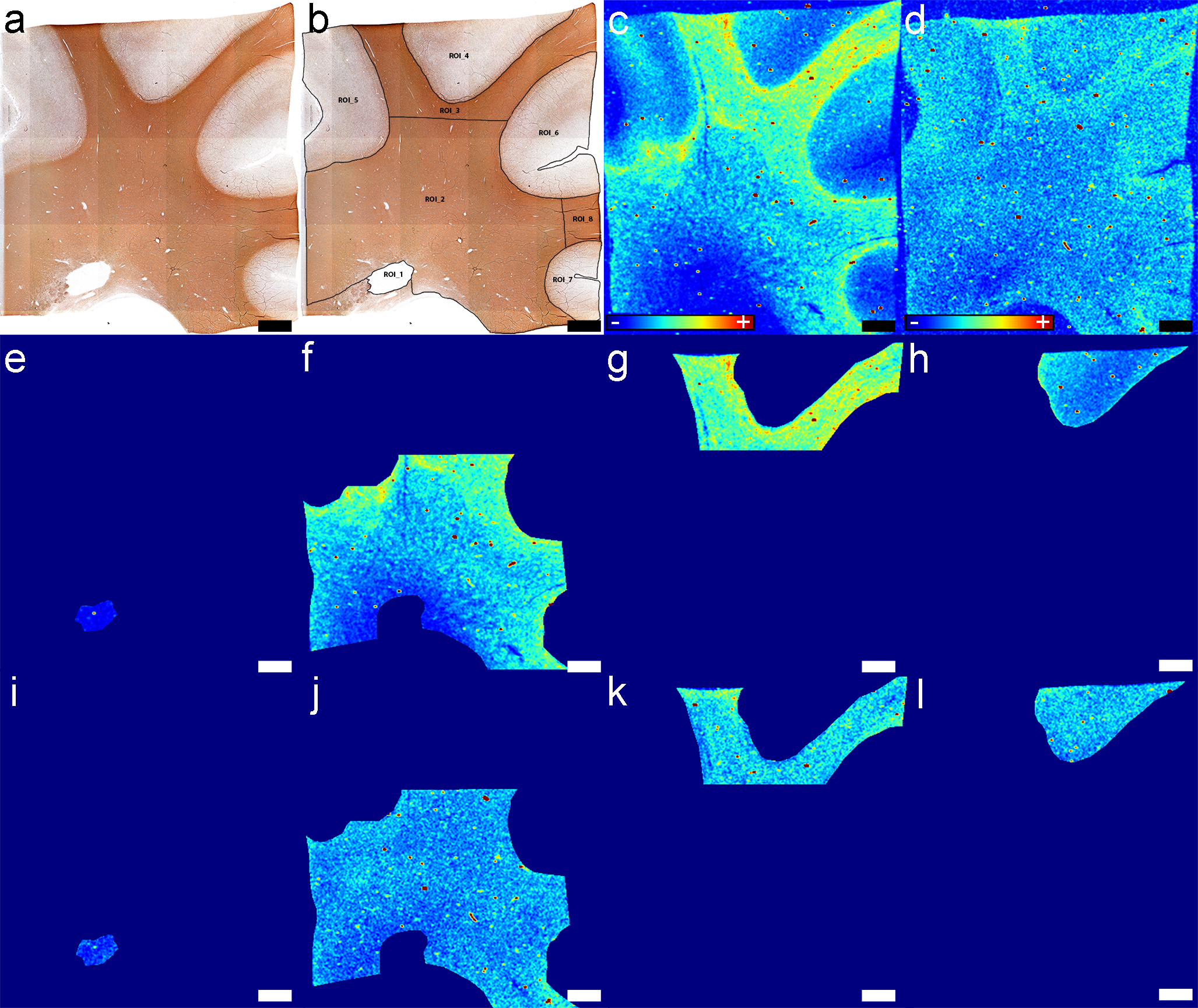

Supplement: Supplementary file 2 — Supplementary material 2 (TIFF 3683 kb) [file 401_2017_1696_MOESM2_ESM.tif]

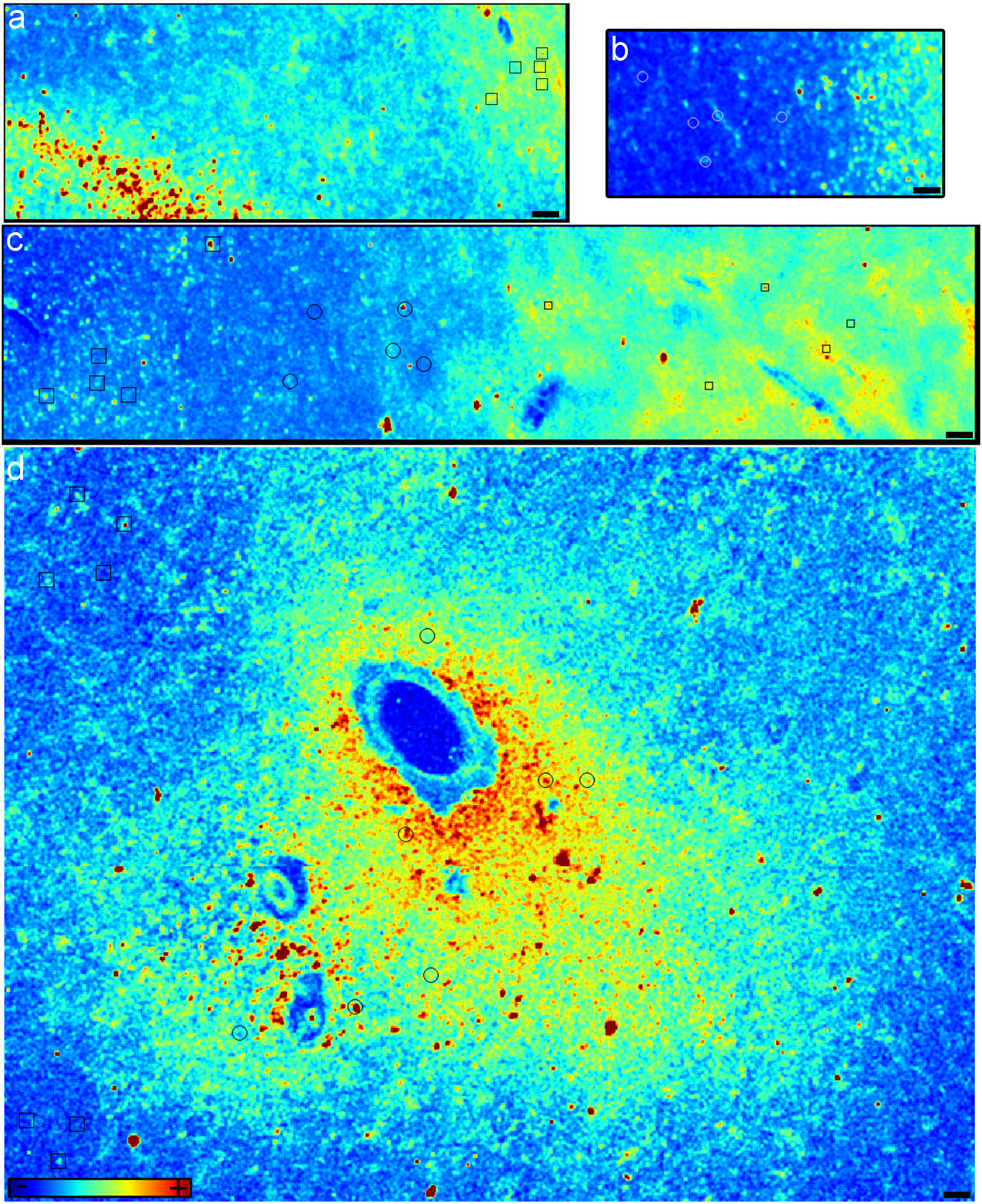

Supplement: Supplementary file 3 — Supplementary material 3 (TIFF 8875 kb) [file 401_2017_1696_MOESM3_ESM.tif]

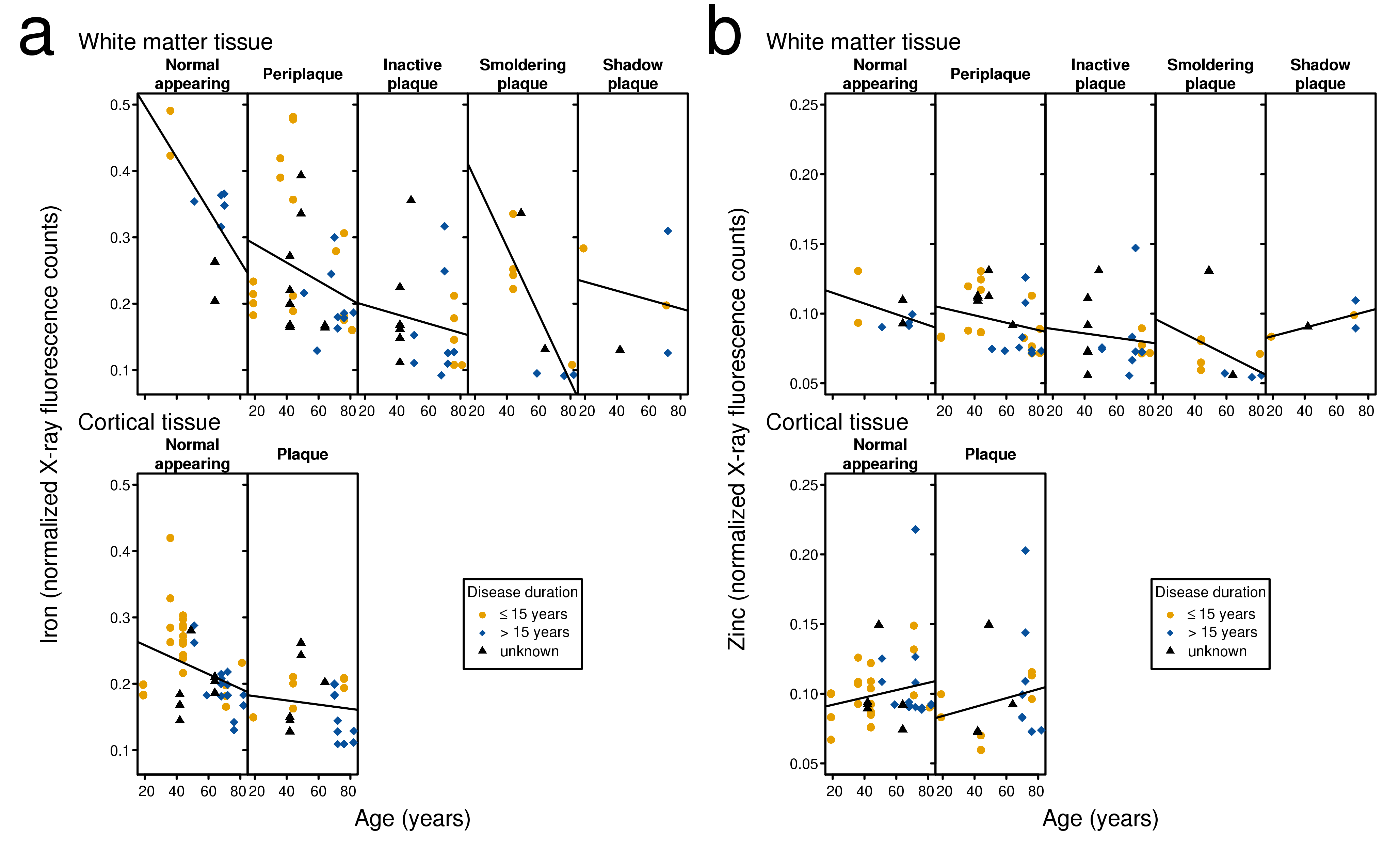

Supplement: Supplementary file 4 — Supplementary material 4 (TIFF 565 kb) [file 401_2017_1696_MOESM4_ESM.tif]

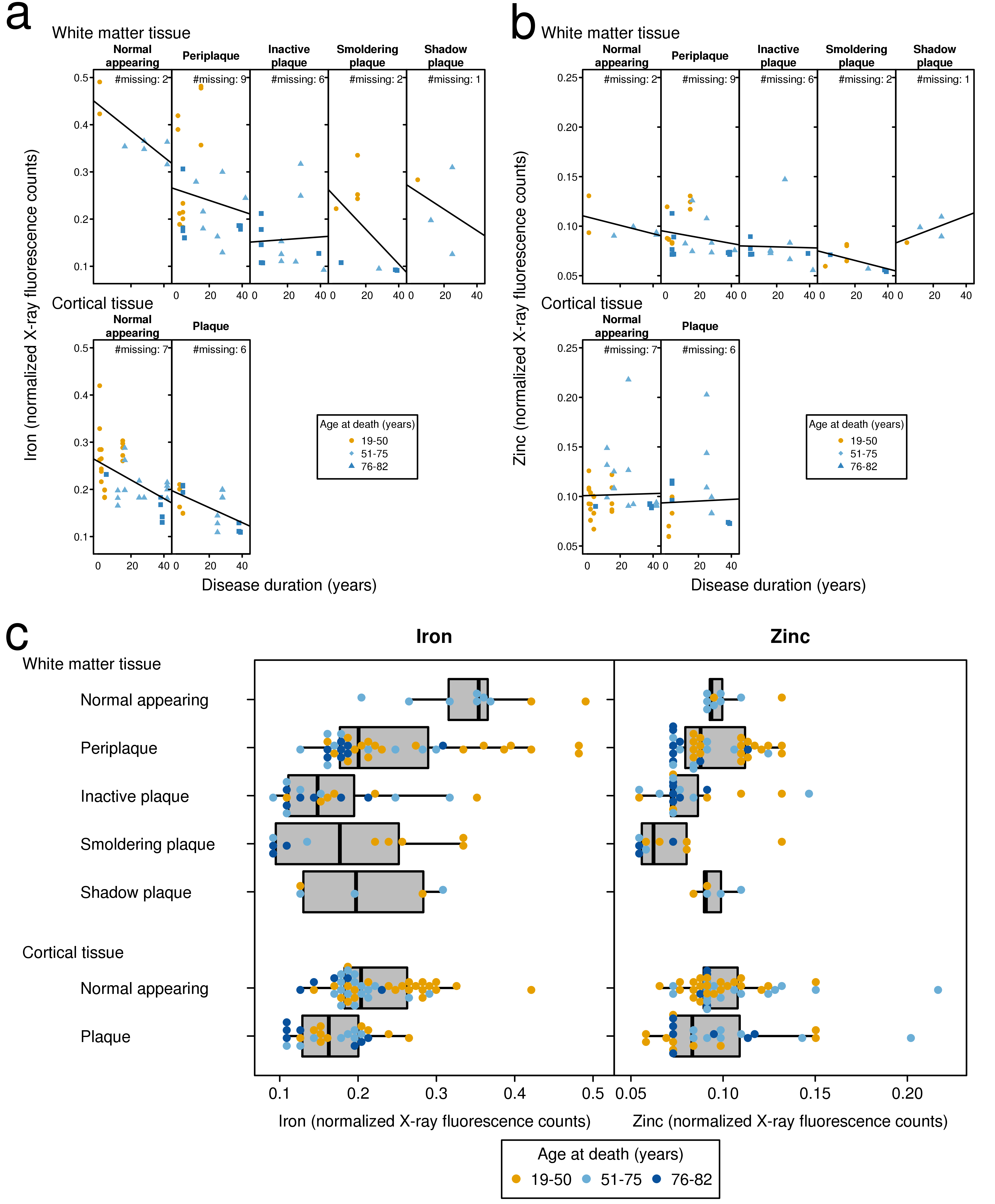

Supplement: Supplementary file 5 — Supplementary material 5 (TIFF 1106 kb) [file 401_2017_1696_MOESM5_ESM.tif]

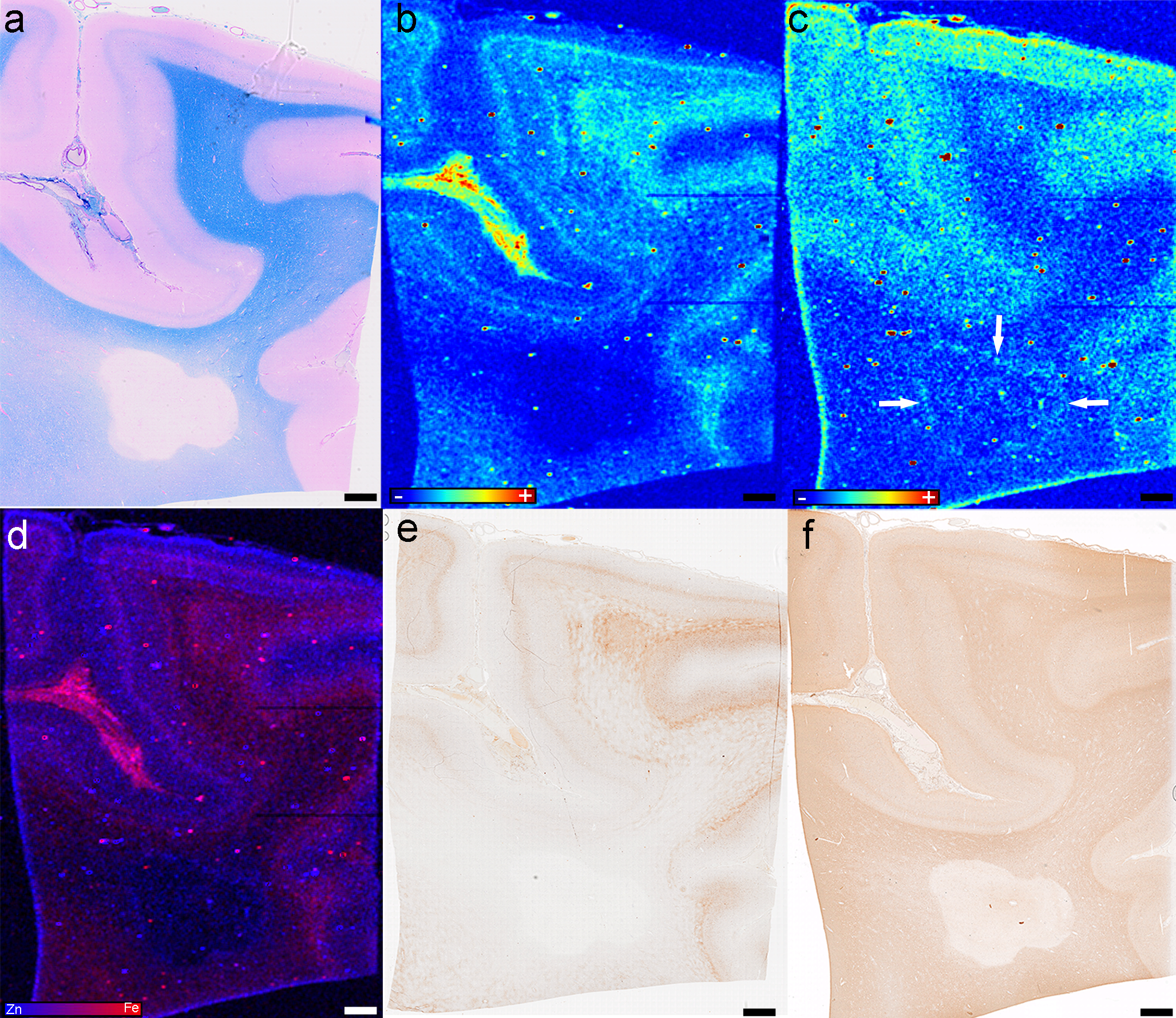

Supplement: Supplementary file 6 — Supplementary material 6 (TIFF 6503 kb) [file 401_2017_1696_MOESM6_ESM.tif]

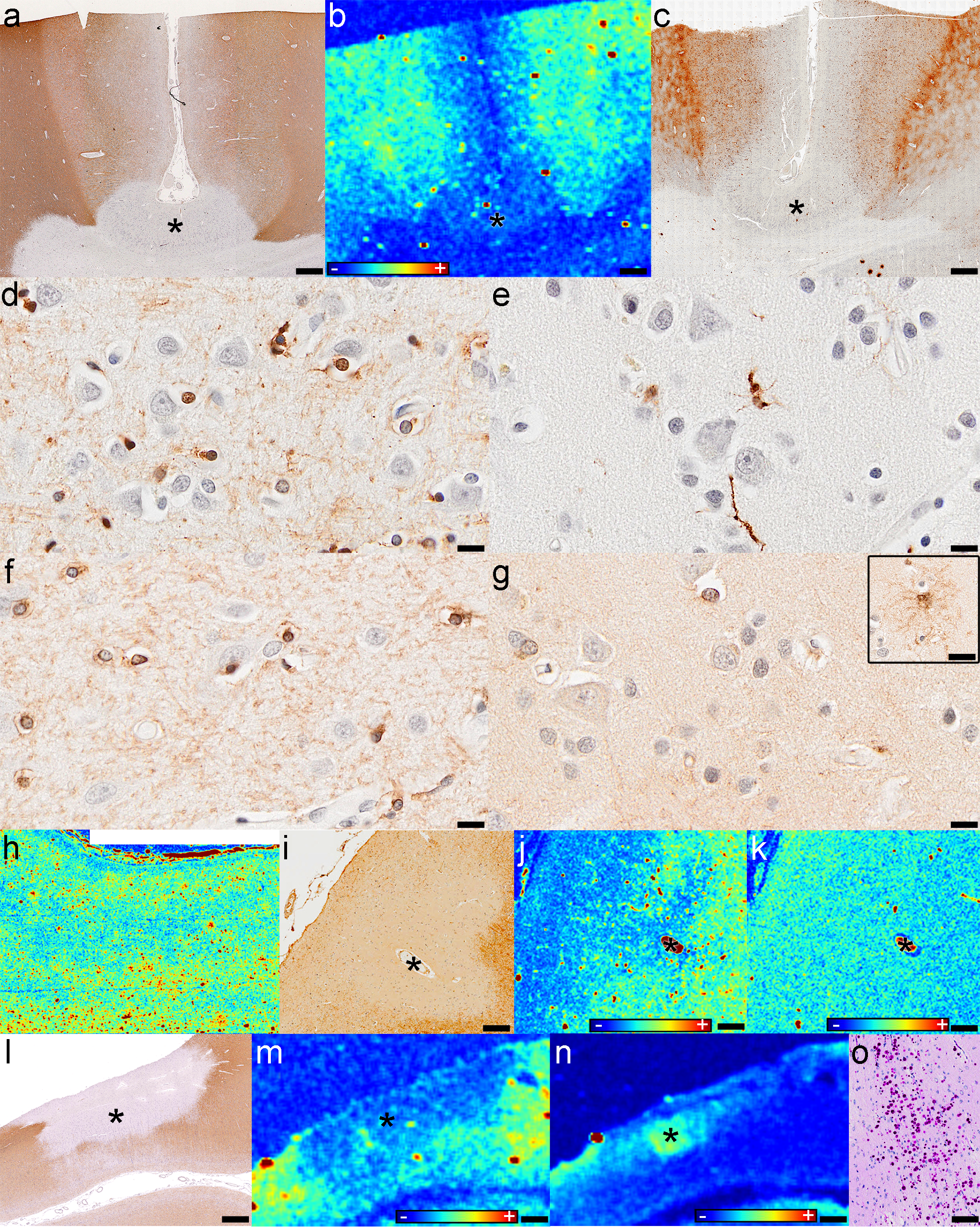

Supplement: Supplementary file 7 — Supplementary material 7 (TIFF 12562 kb) [file 401_2017_1696_MOESM7_ESM.tif]
